# Isolation and prolonged expansion of oral mesenchymal stem cells under clinical-grade, GMP-compliant conditions differentially affects “stemness” properties

**DOI:** 10.1186/s13287-017-0705-0

**Published:** 2017-11-02

**Authors:** Athina Bakopoulou, Danae Apatzidou, Eleni Aggelidou, Evangelia Gousopoulou, Gabriele Leyhausen, Joachim Volk, Aristeidis Kritis, Petros Koidis, Werner Geurtsen

**Affiliations:** 1 Department of Prosthodontics, School of Dentistry, Faculty of Health Sciences, Aristotle University of Thessaloniki (A.U.Th), GR-54124 Thessaloniki, Greece; 2 Department of Preventive Dentistry, Periodontology and Implant Biology, School of Dentistry, Faculty of Health Sciences, Aristotle University of Thessaloniki (A.U.Th), Thessaloniki, Greece; 3 Department of Physiology and Pharmacology, School of Medicine, Faculty of Health Sciences, Aristotle University of Thessaloniki (A.U.Th), Thessaloniki, Greece; 4 cGMP Regenerative Medicine Facility, Department of Physiology and Pharmacology, School of Medicine, Faculty of Health Sciences, Aristotle University of Thessaloniki (A.U.Th), Thessaloniki, Greece; 5 Department of Conservative Dentistry, Periodontology and Preventive Dentistry, Hannover Medical School (MHH), Hannover, Germany

**Keywords:** Oral mesenchymal stem cells, Alveolar bone marrow mesenchymal stem cells, Dental pulp stem cells, Clinical-grade expansion, Good manufacturing practice-compliant cell preparation, Prolonged expansion, “Stemness” properties

## Abstract

**Background:**

Development of clinical-grade cell preparations is central to meeting the regulatory requirements for cellular therapies under good manufacturing practice-compliant (cGMP) conditions. Since addition of animal serum in culture media may compromise safe and efficient expansion of mesenchymal stem cells (MSCs) for clinical use, this study aimed to investigate the potential of two serum/xeno-free, cGMP culture systems to maintain long-term “stemness” of oral MSCs (dental pulp stem cells (DPSCs) and alveolar bone marrow MSCs (aBMMSCs)), compared to conventional serum-based expansion.

**Methods:**

DPSC and aBMMSC cultures (*n* = 6/cell type) were established from pulp and alveolar osseous biopsies respectively. Three culture systems were used: StemPro_MSC/SFM_XenoFree (Life Technologies); StemMacs_MSC/XF (Miltenyi Biotek); and α-MEM (Life Technologies) with 15% fetal bovine serum. Growth (population doublings (PDs)), immunophenotypic (flow cytometric analysis of MSC markers) and senescence (β-galactosidase (*SA-β-gal*) activity; telomere length) characteristics were determined during prolonged expansion. Gene expression patterns of osteogenic (ALP, BMP-2), adipogenic (LPL, PPAR-γ) and chondrogenic (ACAN, SOX-9) markers and maintenance of multilineage differentiation potential were determined by real-time PCR.

**Results:**

Similar isolation efficiency and stable growth dynamics up to passage 10 were observed for DPSCs under all expansion conditions. aBMMSCs showed lower cumulative PDs compared to DPSCs, and when StemMacs was used substantial delays in cell proliferation were noted after passages 6–7. Serum/xeno-free expansion produced cultures with homogeneous spindle-shaped phenotypes, while serum-based expansion preserved differential heterogeneous characteristics of each MSC population. Prolonged expansion of both MSC types but in particular the serum/xeno-free-expanded aBMMSCs was associated with downregulation of CD146, CD105, Stro-1, SSEA-1 and SSEA-4, but not CD90, CD73 and CD49f, in parallel with an increase of SA-gal-positive cells, cell size and granularity and a decrease in telomere length. Expansion under both serum-free systems resulted in “osteogenic pre-disposition”, evidenced by upregulation of osteogenic markers and elimination of chondrogenic and adipogenic markers, while serum-based expansion produced only minor changes. DPSCs retained a diminishing (CCM, StemPro) or increasing (StemMacs) mineralization potential with passaging, while aBMMSCs lost this potential after passages 6–7 under all expansion conditions.

**Conclusions:**

These findings indicate there is still a vacant role for development of qualified protocols for clinical-grade expansion of oral MSCs; a key milestone achievement for translation of research from the bench to clinics.

**Electronic supplementary material:**

The online version of this article (doi:10.1186/s13287-017-0705-0) contains supplementary material, which is available to authorized users.

## Background

In recent years, divergent populations of postnatal mesenchymal stem (stromal) cells (MSCs) have been identified in various tissues, including umbilical cord, bone marrow, adipose and oral tissues [1], and have been extensively studied for their safety and efficacy for numerous clinical applications [2, 3]. MSCs attract worldwide interest for their multilineage differentiation potential into tissue-forming cells having the ability to support repair processes in damaged sites, but more importantly for the paracrine-mediated regenerative effects that they exert through the release of a plethora of trophic and anti-inflammatory/immunosuppressive factors in their secretome, with potential to modulate the host immune responses [4, 5].

Although more than 180 MSC-related clinical trials are currently registered in a database compiled by the US National Institutes of Health (www.clinicaltrials.gov), considerable variation in the mode and procedures of preparing MSCs for clinical use is evident, thus unfolding the urgent and unmet clinical need for establishing universally accepted materials and standard operating procedures (SOPs) for safe, reproducible and economic isolation and large-scale expansion of MSCs at a clinical-grade level. MSCs are currently classified as advanced therapy medicinal products (ATMPs), and therefore are subject to Regulation EC1397/2007 of the European Commission [6]. A recent definition also designates MSCs as cell-based medicinal products (CBMPs); that is, medicinal products presented as having properties for, or used in or administered to, human beings with a view to treating, preventing or diagnosing a disease in which the pharmacological, immunological or metabolic actions are carried out by cells or tissues [7]. These products have to be prepared in compliance with good manufacturing practices (cGMP) that ensure consistent production and controlled quality standards appropriate to their intended use, as described in EU Regulation 2003/94/EC [8]. Such cGMP practices include, among others, several parameters related to MSC isolation and cultivation processes. To specify procedures for proper handling under cGMP conditions, current research focuses on evaluating the effects that expansion procedures might have on the MSC “stemness” characteristics, their phenotypic and genetic stability, the efficacy in regenerating the target tissues and the permitted population doublings (PDs) before senescence emerges to finally establish reliable characterization methodologies for the accurate assessment of each of these parameters [9].

Considerable effort has been devoted to optimizing standard culture protocols. In particular, the use of animal serum as supplementation for conventional culture media is fraught with problems. Although still widely used, its application raises serious concerns related to the highly variable and often unknown composition of each batch, the immunological risks associated with serum proteins and, most importantly, the potential transmission of animal diseases to humans [10]. Serum components in MSC cultures are crucial for effective cell expansion [11] and efforts to replace commercially available bovine or calf serum with cGMP alternatives, such as autologous or allogeneic human sera or human platelet lysate (HPL), have encountered technical difficulties in achieving large-scale expansion of MSCs, and also in meeting the laboratory requirements of these supplements in large amounts [12]. Most recently, the development of cGMP culture systems, free of any animal derivatives (i.e., serum/xeno free), has been achieved by leading life science companies aiming to provide homogeneous, well-defined MSC populations with enhanced “stemness” characteristics, minimized variability and closely controlled MSC functions, meeting the EU regulatory requirements [13].

Another important element for successful implementation of MSC-based therapies is related to selection of the most suitable source to isolate the MSCs, tailored to the intended clinical application [14]. In the field of dental research, various oral MSC sources have been proposed as highly promising for tissue engineering (TE) applications in the orofacial region. Among these, the alveolar crest has been suggested as an advantageous source for alveolar bone marrow-derived MSCs (aBMMSCs) used in maxillofacial bone regeneration [15], as compared to the iliac crest-derived BMMSCs, considered so far to be the “gold standard”. aBMMSCs have been shown to proliferate faster, demonstrate delayed senescence in vitro and form bone in greater amounts in vivo, compared to the iliac crest-derived BMMSCs of the same individuals [16]. An additional benefit of aBMMSCs is the minimally invasive surgical technique required to obtain the osseous biopsies during routine dental procedures (i.e., tooth extraction, implant placement).

Another promising oral MSC source of great importance in regenerative dentistry is the pulp of the adult teeth (dental pulp stem cells (DPSCs)). These cells have been studied extensively for in-vitro multilineage differentiation potential toward osteo/odontogenic, adipogenic, chondrogenic, neurogenic, angiogenic and myogenic lineages [14, 17], while in-vivo studies confirm their ability to reconstitute functional dentin/pulp complexes [18] and other tissues including bone [19], cementum [20], blood vessels [21] and neural tissues [22]. A growing number of “proof of concept” studies validate DPSCs as a very effective MSC source for dental [23] and nondental biomedical applications [24].

In the context of establishing standardized conditions for safe and efficient clinical-grade expansion of oral MSCs, bridging the gap between research models and clinical applications in the orofacial region, the present study aimed to identify the critical “stemness” properties influenced by the culture “micro-milieu” that might have an impact on the MSC clinical performance. The potential of two commercially available serum/xeno-free, cGMP culture systems to maintain the growth and functional properties of two types of oral MSCs (DPSCs and aBMMSCs) was investigated in comparison with a widely and longtime used conventional fetal bovine serum (FBS)-based approach. The growth rates, differentiation potentials and morphological, immunophenotypic and senescence characteristics of MSCs were determined for both cell types after prolonged expansion. The results of this study contribute to the development of qualified protocols and SOPs for clinical-grade expansion of oral MSCs, as a key milestone achievement for translation of research findings to clinical settings.

## Methods

### Establishment of DPSC and aBMMSC cultures

DPSCs (*n* = 6 donors/culture medium under investigation) were established from pulp biopsies of human third molars routinely extracted for orthodontic purposes from young donors aged 18–25 years, while aBMMSC cultures (*n* = 6/culture medium under investigation) were obtained from alveolar osseous biopsies of donors, aged 25–55 years, during routine implant placement procedures. All donors were nonsmokers and systemically healthy. Cell cultures were established by the enzymatic dissociation method, as described previously [25]. Briefly, for DPSC establishment, teeth were disinfected and cut around the cementum–enamel junction to expose the pulp chamber. The pulp tissue was retrieved, minced into small fragments and digested in a solution of 3 mg/ml collagenase type I and 4 mg/ml dispase II (Life Technologies, Thermo Fisher Scientific, Waltham, MA, USA) for 1 h at 37 °C. For aBMMSC establishment, osseous core biopsies extending into the alveolar bone marrow were harvested by means of a rotary trephine bur (2/3 mm diameter on the inside/outside) (BIOMET 3*i*; Impladent Dental Implants, LLC, Thessaloniki, Greece) to a depth of 8–10 mm. Osseous biopsies were then minced using a periodontal bone chisel (36/37, Rhodes, Back Action; Hu-Friedy, ipm dental, Athens, Greece) and subsequently enzymatically digested like the pulp biopsies.

For both types of cultures, cells were placed after enzymatic dissociation of the tissue in 25-cm^2^ flasks (p.0) and incubated at 37 °C in 5% CO_2._ When cultures reached 70–80% confluency, the entire cell population was detached and transferred to a 75-cm^2^ flask (p.1). After reaching 70–80% confluency, cells were detached and subcultured at 5000 cells/cm^2^ in culture plates 100 mm in diameter. Medium change was performed every 2 days and the same process repeated for at least 10 passages. Cells were counted at the beginning and the end of each passage with a hemocytometer (Neubauer cell chamber; Laboroptik, Lancing, UK). MSCs at early (p.2–3), middle (p.6–7) and late (p.10–11) passages were evaluated comparatively.

For MSC expansion, three different culture systems (i.e., two serum/xeno-free, cGMP media and one conventional FBS-based system) were used. Specifically:

1. Conventional medium (Complete Culture Medium (CCM)): comprising alpha Minimum Essential Medium (α-MEM; Life Technologies) supplemented with 15% FBS (Life Technologies), 100 μM l-ascorbic acid phosphate (Sigma-Aldrich, Taufkirchen, Germany) and 1 ml antibiotic solution (100 units/ml penicillin, 100 mg/ml streptomycin) (all LifeTechnologies). At passage completion, cells were detached with a solution of 0.25% trypsin and 1 mM EDTA for 4–5 min and replated at a density of 5000 cells/cm^2^.
2. StemPro MSC SFM XenoFree (“StemPro”; Life Technologies): following the manufacturer’s instructions, 98 ml StemPro MSC SFM Basal Medium was supplemented with 1 ml StemPro Supplement, 1 ml GlutaMAX–I CTS and 50 μl of 50 mg/ml gentamicin reagent solution. Prior to MSC expansion, plastic surfaces were coated with CELLstart CTS (LifeTechnologies) (a recombinant enzyme proposed as a nonanimal alternative to porcine trypsin). At passage completion, cells were detached using TrypLE™ Select CTS™ (LifeTechnologies) (a recombinant enzyme proposed as a nonanimal alternative to porcine trypsin) for 4–5 min and replated at a density of 5000 cells/cm^2^.
3. Miltenyi Stem MACS MSC Expansion Media Kit XF (“StemMacs”; Miltenyi Biotec GmbH, Bergisch Gladbach, Germany): following the manufacturer’s instructions, 98.6 ml StemMACS MSC basal Expansion Media XF was supplemented with 1.4 ml StemMACS MSC Expansion Media XF Supplement and 50 μl of 50 mg/ml gentamicin reagent solution. At passage completion, cells were detached from culture flasks using TrypLE™ Select CTS™ for 4–5 min and replated at a density of 5000 cells/cm^2^.

### Immunophenotypic characterization of DPSC and aBMMSC cultures with flow cytometry

DPSCs and aBMMSCs were characterized for mesenchymal (CD90, CD73, CD49f, CD146, STRO-1), endothelial (CD105), hematopoietic (CD45, CD34, CD117/c-kit) and embryonic (SSEA-1, SSEA-3, SSEA-4) stem cell (SC) markers at p.2–3, p.6–7 and p.10–11 by flow cytometry, as described previously [26], using the following fluorochrome-conjugated mouse anti-human antibodies: CD90-fluorescein isothiocyanate (FITC), CD73-phycoerythrin (PE), CD34-allophycocyanin (APC), STRO-1-FITC, CD146-PE, CD49f-APC, CD105-APC, SSEA-1-PE, SSEA-3-PE, SSEA-4-FITC, CD117-Peridinin-Chlorophyll-Protein-cyanin 5.5 (PerCP-Cy5.5) and CD45-PE (all BioLegend, Fell, Germany). Analysis was performed by means of a Guava® easyCyte 8HT Benchtop Flow Cytometer (Merck Millipore, Billerica, MA, USA). A total of 50,000 events were acquired for each sample. Data were analyzed using GuavaSoft 3.1.1 and Summit 5.1 software. In addition to determining the percentage of cells positive for each marker, the cell size and cell internal complexity (granularity) distribution profiles were analyzed by forward scatter (FSC) vs side scatter (SSC) fluorescence intensity plots, respectively.

### Evaluation of morphological characteristics and cell proliferation

The proliferative potential of oral MSCs was assessed by estimating the population doubling time (PDT) over passages. The formula 2^*n*^ = *Nx* / *No* was used to calculate the PDs of cells at each passage based on the number of cells counted with a hemocytometer after detachment (*Nx*) and the number of cells initially plated (*No*). PD is then determined as PD = log_2_(*Nx* / *No*). At passage 0 the total number of cells initially attached (after 48 h) to the culture flasks was counted and used as the initial *No*. Cumulative PDs were also calculated as described previously [12]. Finally, the average PDT for each passage was calculated as *t* / *n*, where *t* is the duration of culture in days and *n* is the number of PDs reached during passage, calculated as described.

Cell morphology was visualized under a phase-contrast microscope (Zeiss Axiovert 40; Carl Zeiss micro imaging, GmbH, Göttingen, Germany) equipped with a digital camera with appropriate software (Carl Zeiss Axiovision 4.6 software). Pictures of randomly chosen areas were taken, in order to reflect representative growth patterns.

### Evaluation of oral MSC senescence

#### Senescence-associated β-galactosidase assay

Expression of senescence-associated β-galactosidase (SA-β-gal) at p.2–3, p.6–7 and p.10–11 was determined by a chromogenic assay kit (Sigma-Aldrich), according to the manufacturer’s instructions. Briefly, cells, were fixed in 4% PFA, and then washed with PBS and incubated with β-Gal staining solution (40 mM citric acid sodium phosphate buffer, 1 M NaCl, 5 mM ferrocyanide, 5 mM ferricyanide, 2% DMF, 20 mM MgCl_2_, X-GAL 1 mg/ml in DMSO) for 14–16 h at 37 °C. Stained and unstained cells were counted under a light microscope in six randomly selected optical fields of vision (×100) and the percentage of positive cells was calculated. Blinded subjective scoring of the percentage of blue-stained cells was used to quantify senescent cell fractions.

#### Evaluation of MSC relative telomere length measurement

Purified genomic DNA (gDNA) was extracted using the Nucleospin® Tissue DNA isolation kit (Macherey Nagel, Düren, Germany). To evaluate the relative telomere length of different cells, passages and expansion media, the TeloTAGGG Telomere Length Assay Kit (Roche, Indianapolis, IN, USA) was used. Following the kit protocol, 2 μg of gDNA/sample was first double-digested with *Hin*fI and *RSA*I and subjected to electrophoresis on a 1% agarose/TAE gel, and then Southern blot-transferred onto a positively charged nylon membrane by overnight capillary blot in 20× standard sodium citrate (SSC) buffer. The gDNA on the nylon membrane was then hybridized with a digoxigenin (DIG)-labeled probe, incubated with a DIG-specific antibody covalently coupled to alkaline phosphate and visualized using chemiluminescent substrate according to the manufacturer’s protocol. Membranes were exposed to an enhanced chemiluminescence (ECL) imaging system (MicroChemi; DNR Bio-Imaging Systems Ltd, Neve Yamin, Israel).

Telomere lengths were determined by analysis of terminal restriction fragments (TRFs) calculated as follows:

$$ \mathrm{TRFs}=\Sigma (ODi)/\Sigma \left( ODi/ Li\right) $$

where *ODi* is the chemiluminescent signal and *Li* is the length of the TRF at position *i*. The calculation accounts for the higher signal intensity from larger TRFs due to multiple hybridizations of the telomere-specific hybridization probe. TeloTool software [27, 28] was used for TRF analysis.

### Evaluation of time-course mRNA expression of osteogenic, adipogenic and chondrogenic markers

Quantitative real-time polymerase chain reaction (qPCR) was used to assess spontaneous upregulation or downregulation of osteogenic (alkaline phosphatase (ALP), bone morphogenetic protein 2 (BMP-2)), adipogenic (lipoprotein lipase (LPL), peroxisome proliferator-activated receptor gamma (PPAR-γ)) and chondrogenic (aggrecan (ACAN), transcription factor SOX-9) markers during prolonged culture with each of the culture systems under investigation. Total mRNA was isolated from DPSCs and aBMMSCs at p.2–3, p.6–7 and p.10–11 using the Nucleospin RNA isolation kit (Macherey Nagel) and was reverse-transcribed (1 μg/sample) using the superscript first-strand synthesis kit (Invitrogen), according to the manufacturer’s instructions. Reactions were performed using SYBR-Select PCR Master Mix (Applied Biosystems, Foster City, CA, USA) in a Step One Plus thermal cycler (Applied Biosystems). All reactions started with two incubation steps at 50 °C for 2 min for uracil-*N*-glycosylase (UNG) activation and at 95 °C for 2 min for activation of the AmpliTaq DNA polymerase. This was followed by 40 cycles of PCR, comprising denaturation for 15 sec at 95 °C and annealing/extension for 1 min at 60 °C. Primers for these genes were designed using an online primer design tool (www.ncbi.nlm.nih.gov) (Table 1). The results were adjusted by amplification efficiency (LinRegPCR) and normalized against two housekeeping genes (succinate dehydrogenase complex, subunit A, flavoprotein (SDH-A) and beta-2-microglobulin (B2M)), and found to remain stable during differentiation processes of these cells, as evaluated by geNorm.

**Table 1:** Primers designed for real-time PCR and the respective amplicon sizes of the products

Gene symbol	Type of marker	Forward (5′–3′)	Reverse (5′–3′)	Amplicon size (bp)
*BMP-2*	Osteogenic	GGAACGGACATTCGGTCCTT	AGTCCGTCTAAGAAGCACGC	100
*ALP*	Osteogenic	CCGTGGCAACTCTATCTTTGG	CAGGCCCATTGCCATACAG	89
*RUNX2*	Osteogenic	CCACCGAGACCAACAGAGTC	TCACTGTGCTGAAGAGGCTG	118
*BGLAP*	Osteogenic	GACTGTGACGAGTTGGCTGA	AAGAGGAAAGAAGGGTGCCT	137
*ACAN*	Chondrogenic	CACCTCCCCAACAGATGCTT	GGTACTTGTTCCAGCCCTCC	107
*SOX9*	Chondrogenic	AGGAAGTCGGTGAAGAACGG	CGCCTTGAAGATGGCGTTG	84
*PPAR-γ*	Adipogenic	GACAACCTGCTACAAGCCCT	TTGGCAAACAGCTGTGAGGA	71
*LPL*	Adipogenic	CGAGCGCTCCATTCATCTCT	CCAGATTGTTGCAGCGGTTC	137
*B2M*	Housekeeping	TGTCTTTCAGCAAGGACTGGT	ACATGTCTCGATCCCACTTAAC	138
*SDHA*	Housekeeping	GCATGCCAGGGAAGACTACA	GCCAACGTCCACATAGGACA	127

### Evaluation of the in-vitro osteogenic and chondrogenic differentiation potential

In this last set of experiments, DPSCs and aBMMSCs at p.2–3, p.6–7 and p.10–11 were induced to differentiate toward osteogenic and chondrogenic lineages using respective differentiation inductive media, to assess potential loss of MSC functionality after prolonged expansion with each culture system under investigation. Adipogenic differentiation is of limited interest in TE and was therefore not further assessed. Specifically, for this assay, early, middle and late-passage DPSCs and aBMMSCs of each culture medium group were seeded at a density of 3 × 10^5^ cells/well on six-well plates. After 2–3 days, confluent MSCs were exposed to either osteogenic or chondrogenic media according to the manufacturer’s instructions (StemPro Osteogenic and StemPro Chondrogenic Differentiation Kits; Life Technologies). After 7 and 14 days, cell lysates were processed for RNA extraction to perform qPCR (as already described) and evaluate expression of osteogenic (ALP, BMP-2, bone gamma-carboxyglutamate protein/osteocalcin (BGLAP)) and chondrogenic (ACAN, SOX-9) markers. Additionally, since bone formation remains a major target tissue in craniofacial regeneration, cells exposed to osteogenic medium for 21 days were histochemically stained with 1% Alizarin Red S, followed by quantification of mineral content by the cetylpyridinium chloride (CPC) extraction method, as described previously [25]. Optical density (OD) was measured at 550 nm using a microplate reader (Epock; Biotek Instruments, Inc., VT, USA).

### Statistics

All experiments were run in two to four replicates and repeated at least three times. Statistical analysis of the data employed one-way or two-way analysis of variance (ANOVA), while multiple comparisons between groups (i.e., DPSCs vs aBMMSCs; CCM vs StemMacs vs StemPro; and early vs middle vs late passages) for each of the biological endpoints under investigation were performed with Tukey’s post-hoc test, using Prism 6.0 software (GraphPad, CA, USA). Normal distribution was confirmed by D’Agostino and Pearson normality tests. Data were expressed as mean (standard deviation (SD)).

## Results

### Growth patterns and morphological characteristics

**Fig. 1:**
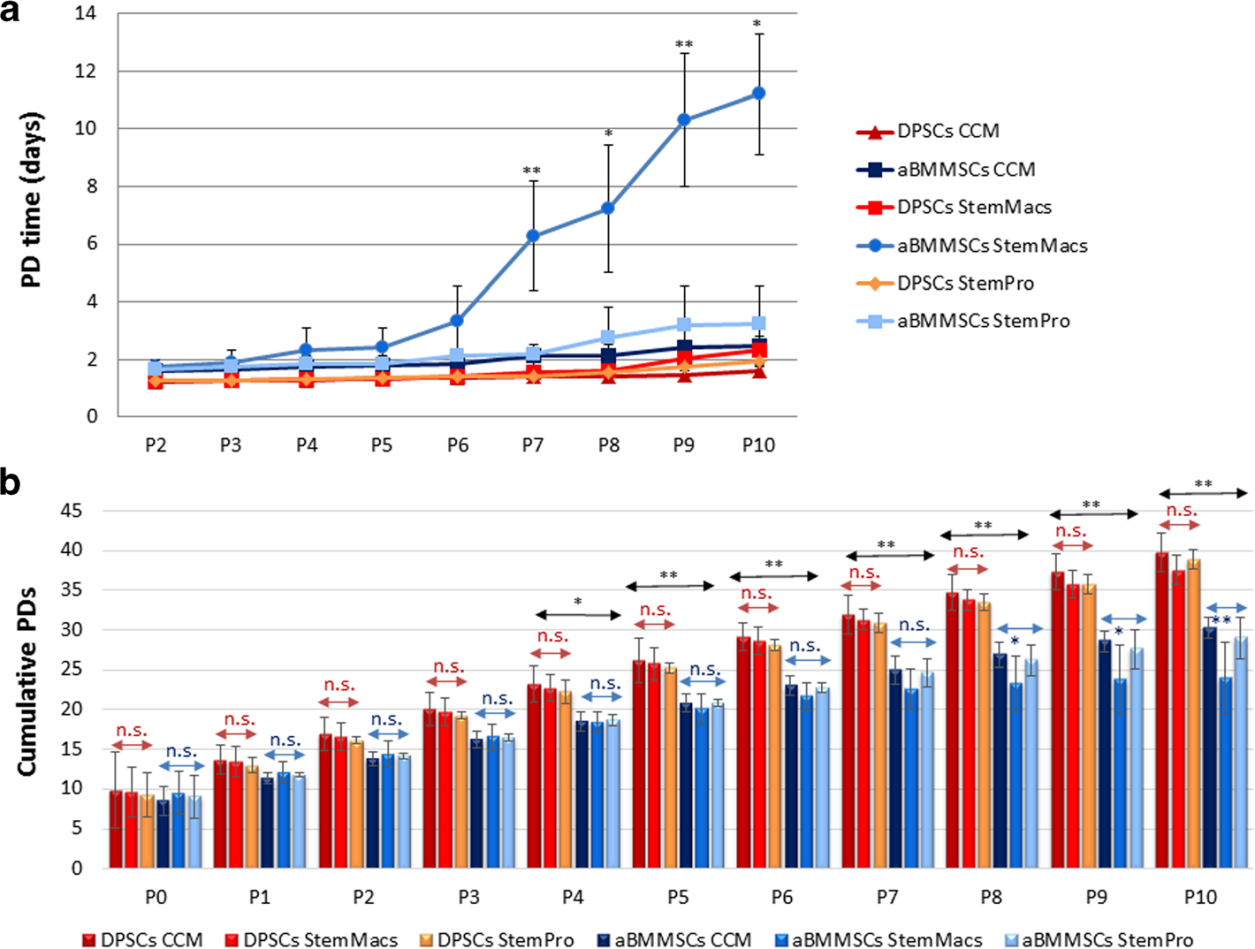
Growth characteristics of DPSCs and aBMMSCs after long-term expansion with three different culture media: one serum-based (CCM) and two serum/xeno-free, cGMP media (StemMacs and StemPro). **a** Population doubling (PD) time (days) (mean ± SD) for DPSCs (*n* = 6 donors, experiments repeated three times in duplicates) and aBMMSCs (*n* = 4 donors, experiments repeated three times in duplicates) through passages. Comparisons between cells, passages and media performed by two-way ANOVA followed by Tukey’s post-hoc tests. Asterisks indicate statistically significant increase in PDT at consecutive passages of each culture medium/cell type (*p < 0.05; **p < 0.01). **b** Total cumulative PD numbers (mean ± SD) for DPSCs (*n* = 6) and aBMMSCs (*n* = 4) calculated based on the ratio of cells seeded vs cells harvested per passage. Asterisks indicate statistically significant differences (*p < 0.05; **p < 0.01; n.s. = nonsignificant, calculated by two-way ANOVA followed by Tukey’s post-hoc tests) either among the three different media (depicted below/above red or blue double arrows for DPSCs and aBMMSCs, respectively) or between the two different cell types (over horizontal black double arrows). aBMMSC alveolar bone marrow mesenchymal stem cell, CCM complete culture medium, DPSC dental pulp stem cell, P cell passage

Comparative analysis was performed to assess the ability of three different culture systems (one conventional serum-based and two serum/xeno-free cGMP approaches) to support DPSC and aBMMSC initial culture establishment from pulp and osseous biopsies, respectively, and subsequent cell expansion up to p.10. Figure 1a shows the PDT (in days) of each cell type/culture condition from p.2 onward up to p.10; Fig. 1b illustrates the cumulative PD numbers from initial culture establishment (p.0) up to p.10.

Calculation of the PDT at each passage (starting with the same plating density of 5000 cells/cm^2^ for all cell types/culturing conditions and counting cell numbers at the beginning/end of each passage) showed no statistically significant increase in PDT up to p.10 in CCM, StemMacs and StemPro-expanded DPSCs (*p* ≥ 0.9997, *p* ≥ 0.8158 and *p* ≥ 0.9815, respectively) and in CCM and StemPro-expanded aBMMSCs (*p* ≥ 0.9157 and *p* ≥ 0.2992, respectively) (Fig. 1a). In contrast, aBMMSCs expanded with StemMacs showed no significant increase in PDT up to p.6 (*p* ≥ 0.2658), but from p.7 onward significant delays in cell growth were observed (*p* < 0.0001 for p.6 vs p.7; *p* = 0.0312 for p.7 vs p.8; *p* < 0.0001 for p.8 vs p.9; *p* = 0.0323 for 9 vs 10; Fig. 1a). This was evidenced by substantially increased PDTs of StemMacs-expanded aBMMSCS, as compared with those expanded with the other two culture media. In two out of six StemMacs-expanded aBMMSC cultures, cells proliferated extremely slowly after p.7.

The initial time required from biopsy uptake to p.1 (performed at 70–80% cell confluency of p.0) was 10.3 (2.1), 10.9 (3.2) and 9.2 (1.9) days for CCM, StemMacs and StemPro-expanded DPSCs, respectively, with no significant differences across culture medium groups (*p* ≥ 0.7558), indicating in general similar isolation efficiency potential. The respective values for aBMMSCs were 13.4 (3.2), 14.3 (3.9) and 12.9 (2.5) days, failing to reach statistical significance for any of the media used (*p* ≥ 0.8258). The time (in days) for reaching p.1 was overall significantly lower for DPSCs compared to aBMMSCs for all culture media (*p* = 0.0279). It should be noted that some (1/6 for CCM, 2/6 for StemMacs and 1/6 for StemPro) aBMMSC cultures failed to reach p.1 after 18 days, due to the low initial cell yield or the very slow growth rate, and were thus excluded from the study.

Regarding cumulative PD numbers (Fig. 1b) no significant differences were found among the DPSCs expanded under the three different culturing systems at any passage (*p* ≥ 0.6969). After 10 passages, DPSCs reached 39.8 (2.44), 37.6 (1.90) and 38.9 (1.28) cumulative PDs for CCM, StemMacs and StemPro-expanded cells, respectively; these being completed after 46.3 (2.1), 46.9 (3.2) and 45.2 (1.9) days, correspondingly. In contrast, aBMMSC groups showed no significant differences (*p* ≥ 0.4971) in cumulative PDs up to p.7, whereas from p.8 onward StemMacs-expanded aBMMSCs showed significantly lower cumulative PDs, compared to those expanded with CCM (*p* < 0.0001 at p.10) or StemPro (*p* = 0.0026 at p.10) (Fig. 1b). After 10 passages, aBMMSCs reached 30.2 (1.36), 23.9 (6.5) and 28.9 (2.64) cumulative PDs for CCM, StemMacs and StemPro-expanded aBMMSCs, respectively; these being completed 49.4 (3.2), 66.3 (7.6) and 48.9 (2.5) days following initial seeding. No substantial differences in cumulative PD numbers were found between DPSC and aBMMSC cultures up to p.3, whereas from p.4 onward all DPSC culture media groups had significantly higher cumulative PD numbers compared to all respective aBMMSC groups (*p* values at each passage are shown in Fig. 1b).

Another important observation was that the methodology presented in this study for initial culture establishment and subsequent cell expansion is able to produce a cell yield of approximately 30 million DPSCs after completion of p.2 and approximately 1 billion DPSCs (if the expansion continues without discarding any part of the population) after completion of p.3; the respective values for aBMMSCs are 10 million and 30 million, respectively.

**Fig. 2:**
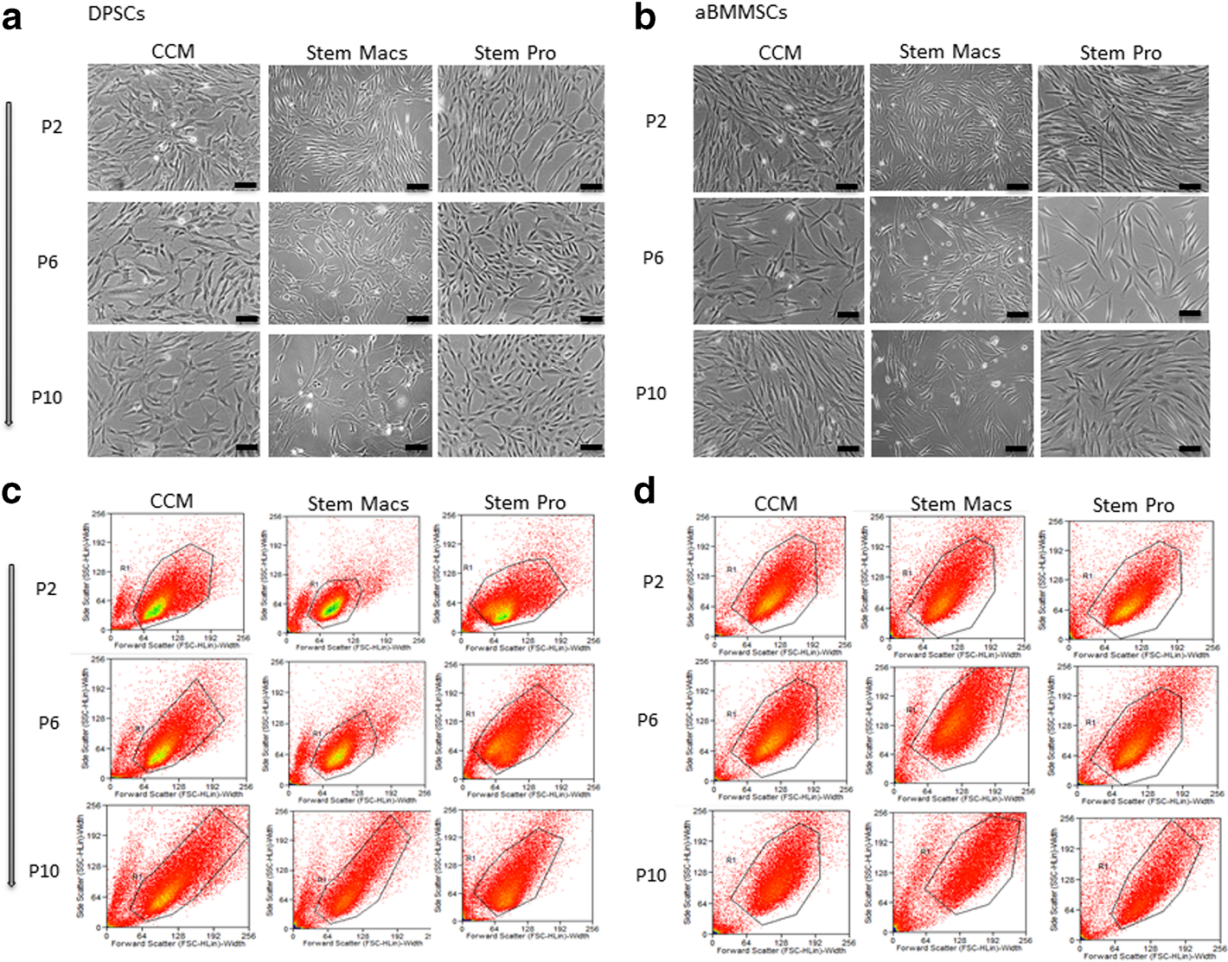
Morphological characteristics of DPSCs and aBMMSCs after long-term expansion with three different culture media: one serum-based (CCM) and two serum/xeno-free, cGMP media (StemMacs and StemPro). **a, b** Phase-contrast microscopy photographs of DPSCs and aBMMSCs, respectively (sale bars: 100 μm). **c, d** Flow cytometry fluorescence intensity plots of forward scatter (FSC) vs side scatter (SSC) parameters corresponding to the cell size and cell internal complexity (granularity), respectively. aBMMSC alveolar bone marrow mesenchymal stem cell, CCM complete culture medium, DPSC dental pulp stem cell, P cell passage

Evaluation of cell morphological characteristics under phase-contrast microscopy (Fig. 2a, b) revealed that serum-expanded DPSCs and aBMMSCs presented noticeable population heterogeneity, consisting of spindle-shaped to stellate-like cells of different sizes, with protrusions of varying number and length; this diversity in phenotype was evident up to late passages. Overall, DPSC cultures consisted of cells considerably smaller in size compared to aBMMSCs; however, they contained several larger cells, seen both at early and late passages, possibly indicating that an intrinsic heterogeneity exists in the cell population. In contrast, DPSC and aBMMSC cultures expanded with both serum-free systems showed a very homogeneous phenotype comprising well-aligned, slender and spindle-shaped cells. This morphology, however, was not maintained at late passages, where a high proportion of flattened, senescent-like cells with multiple intracellular filaments became evident. This was mostly prominent in StemMacs-expanded aBMMSC cultures (Fig. 2b), in accordance with the growth/kinetics data (Fig. 1a, b)

Flow cytometric analysis of cell size vs cell internal complexity (granularity) distribution profiles (FSC vs SSC fluorescence intensity plots; Fig. 2c, d) showed a progressive increase in cell size and granularity with passaging in all types of cultures, but more pronounced in StemMacs-expanded aBMMSC cultures (Fig. 2d), also in conformity with the growth/kinetics data (Fig. 1a, b).

### Immunophenotypic profiles

**Fig. 3:**
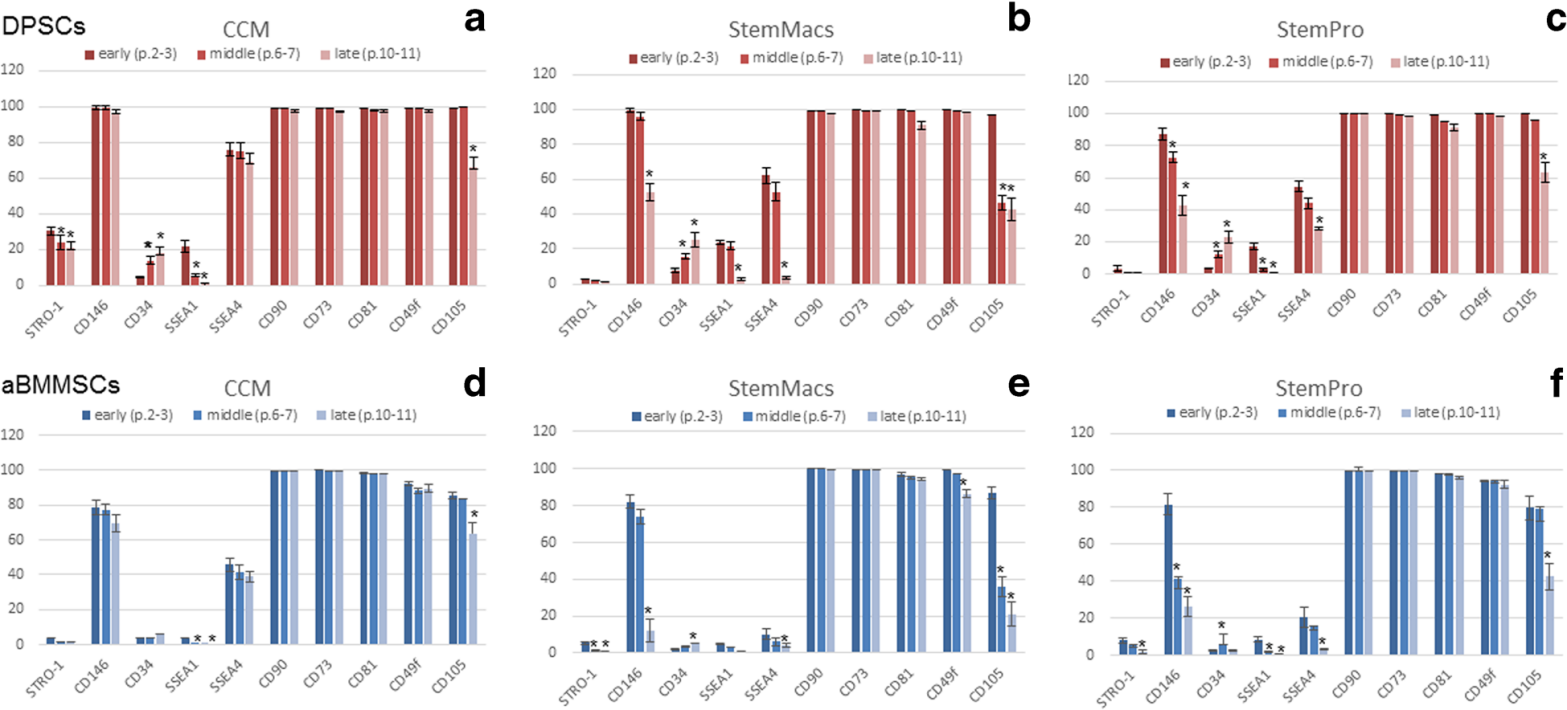
Flow cytometric analysis of several mesenchymal (STRO-1, CD146, CD34, CD90, CD73, CD81, CD49f, CD105) and embryonic (SSEA-1, SSEA-4) markers for each expansion medium at early, middle and late passages. Values are mean (± SD) of three independent experiments of a single representative DPSC (**a–c**) and aBMMSC (**d–f**) culture. *p < 0.05. aBMMSC alveolar bone marrow mesenchymal stem cell, CCM complete culture medium, DPSC dental pulp stem cell, p. cell passage

Figure 3 demonstrates representative findings of a single DPSC and aBMMSC culture for each expansion medium at early, middle and late passages. Additional file 1 contains a full panel of representative flow cytometry diagrams of these markers in DPSC cultures at early, middle and late passages.

Immunophenotypic analysis of DPSC (*n* = 6 donors/culture medium) and aBMMSC (*n* = 4 donors/culture medium) cultures at p.2–3, p.6–7 and p.10–11 revealed that both types of cells, expanded under either serum-based or serum-free conditions, exhibited very high expression (>95% of the population) of the MSC markers CD90, CD73, CD81 and CD49f/a6-integrin at early passages that was maintained unaltered (CD90, CD73) or slightly but not statistically significantly downregulated (CD81, CD49f) at late passages. These findings were consistent for all cell donors (*n* = 6/culture system for DPSCs; *n* = 4/culture system for aBMMSCs) and all three expansion systems (CCM, StemMacs, StemPro). Other markers such as CD105 (>90% DPSCs; > 80% aBMMSCs) and CD146 (>80% DPSCs; > 70% aBMMSCs) were also highly expressed at early passages, but were substantially downregulated at the middle or late passages depending on cell type and donor. Noteworthy, downregulation of these markers was significantly more prominent at the middle to late passages of serum-free (StemMacs, StemPro) expanded cultures, and of smaller magnitude in the serum-based expansion of both DPSCs and aBMMSCs.

Less abundant expression was observed at early passages (p.2–3) for the MSC marker STRO-1 (15.2 (17.7), 4.2 (2.1) and 5.6 (3.2)% for DPSCs and 3.6 (2.3), 3.2 (1.4) and 7.6 (3.8)% for aBMMSCs expanded with CCM, StemMacs and StemPro, respectively) and the embryonic markers SSEA-1 (21.9 (5.3), 23.6 (3.2) and 18 (4.2)% for DPSCs and 3.7 (1.1), 5.2 (1.3) and 7.3 (2.8)% for aBMMSCs expanded with CCM, StemMacs and StemPro, respectively) and SSEA-4 (69.1 (10.6), 52.2 (13.2) and 55.3 (11.3)% for DPSCs and 45.5 (9.6), 20.2 (8.2) and 32.6 (9.8)% for aBMMSCs expanded with CCM, StemMacs and StemPro, respectively). The aforementioned values represent means of six DPSC and four aBMMSC donors evaluated in three independent experiments for each donor. Of paramount importance was that some of the aforementioned markers (CD146, CD105, STRO-1, SEEA-1, SSEA-4) were substantially downregulated with passaging, but more pronounced in the serum-free compared to the serum-based expanded DPSC and aBMMSC cultures (except SSEA-1 that showed similar trends in all cultures). Despite the interindividual variations observed in total % expression of these markers, these patterns were consistent for both cell types and all three expansion media, as well as in all donor biopsies used for cell culture establishment.

The initial very low (<5%) expression of CD34 found at early passages in most DPSC and aBMMSC cultures increased gradually during prolonged culture expansion. Although this finding was inconsistent with all cell types/donors/media, it was noted in several cultures and seemed to be donor related; the latter requiring further investigation. Finally, the hematopoietic stem cell markers CD45 and CD117/c-kit and the embryonic marker SSEA-3 were not expressed (<1%) in any cell types/donors/media during the entire expansion period.

Statistical analysis of these data revealed that serum (CCM)-expanded DPSCs at early passages showed an overall significantly higher expression of STRO-1 (*p* > 0.001) and SSEA-4 (*p* > 0.001) but not of SSEA-1 compared to StemMacs and StemPro-expanded DPSCs, with no substantial differences between the latter two. On the other hand, significant medium-related differences were observed in aBMMSC cultures for SSEA-4 (CCM > StemPro, *p* > 0.001; and StemPro > StemMacs, *p* > 0.05 in most cell donors) but not for STRO-1 and SSEA-1 expression, the latter two being overall minimally expressed. Finally, when comparing the two cell types, higher SSEA-1 (*p* > 0.001) and SSEA-4 (*p* > 0.001) expressions were recorded for DPSC cultures expanded with all three media, as compared to the respective aBMMSC cultures, while substantially higher STRO-1 expression was observed in CCM-expanded DPSCs (15.2 ± 17.7, with, however, very high variability observed among different cell donors, as evidenced by the high SD), compared with all other cell and media groups, where STRO-1 expression was overall quite low (in the range of 2–8%).

### Senescence and telomere length analysis

**Fig. 4:**
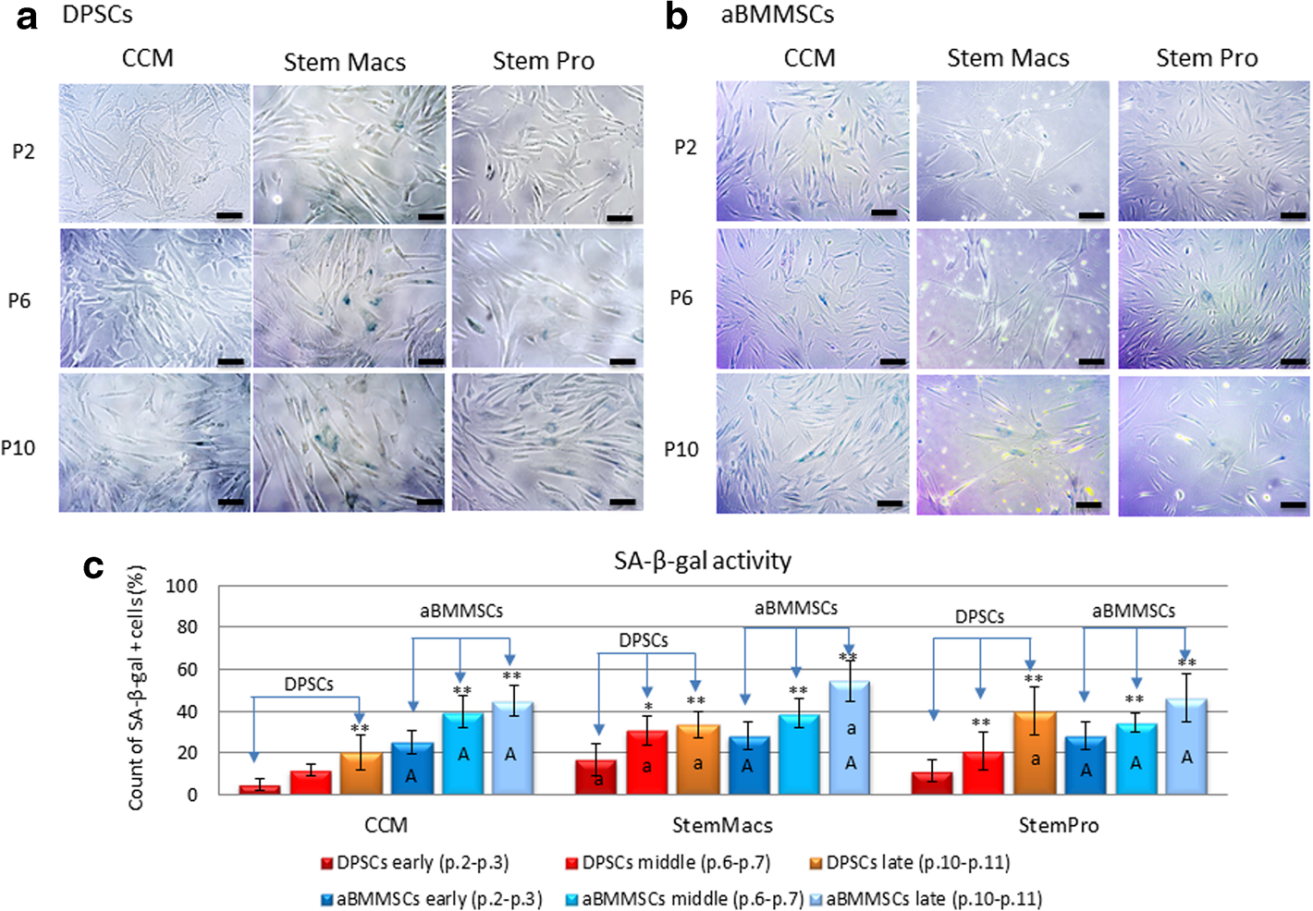
Impact of long-term expansion of DPSCs and aBMMSCs using three different culture media (CCM, StemMacs and StemPro) on β-galactosidase activity. **a, b** Optical microscopy photographs of DPSCs and aBMMSCs, respectively (sale bars: 100 μm). **c** Percentage of SA-β-gal-positive cells (DPSCs and aBMMSCs) at early, middle and late passages of each expansion medium. Values are mean (± SD) of DPSCs (*n* = 6 donors, experiments repeated three times in duplicates) and aBMMSCs (*n* = 4 donors, experiments repeated three times in duplicates). Asterisks indicate statistically significant differences (*p < 0.05; **p < 0.01) between middle vs early and late vs early passages for each cell type and medium separately. Upper case letters (A) indicate statistically significant differences between DPSCs and aBMMSCs expanded with the same medium (either CCM, StemMacs or StemPro) at each passage separately (either early, middle or late), while lowercase letters (a) indicate statistically significant differences between StemMacs vs CCM and between StemPro vs CCM for each cell type (either DPSCs or aBMMSCs) and each passage (either early, middle or late) separately. Statistical analyses were performed by two-way ANOVA followed by Tukey’s post-hoc tests. aBMMSC alveolar bone marrow mesenchymal stem cell, CCM complete culture medium, DPSC dental pulp stem cell, P cell passage, SA-β-gal beta-galactosidase

To investigate whether the observed differences in cell growth rates, immunophenotypic profiles and morphological characteristics were associated with replicative cell senescence (as an indication of defective proliferative capacity), the SA-β-gal activity in DPSC and aBMMSC cultures was evaluated after prolonged expansion under serum-based and serum-free conditions (Fig. 4a–c). A significant increase of SA-β-gal-positive cells with increasing cell passage was observed for both cell types (*p* > 0.001) and all three-expansion media (*p* > 0.001). It was also shown that the number of SA-β-gal-positive cells was significantly higher for aBMMSCs compared to DPSCs at early (*p* < 0.001 for CCM, *p* = 0.0287 for StemMacs and *p* = 0.0001 for StemPro), middle (*p* < 0.001 for CCM, *p* = 0.3957 for StemMacs and *p* = 0.0087 for StemPro) and late (*p* < 0.001 for CCM, StemMacs and StemPro) passages. These results were statistically significant with only the exception of StemMacs-expanded cells at the middle passages, where both aBMMSC and DPSC cultures showed similar, relatively high numbers of SA-β-gal-positive cells. Furthermore, DPSCs expanded with StemMacs (at early (*p* = 0.0122), middle (*p* < 0.0001) and late (*p* = 0.0015) passages) and with StemPro (only at late passages, *p* < 0.0001) demonstrated a significantly higher number of SA-β-gal-positive cells compared to those expanded with the serum-based CCM. In contrast, no major differences were observed for aBMMSCs expanded with the three different media, except for those expanded with StemMacs showing significantly higher numbers of SA-β-gal-positive cells at late passages (*p* < 0.0001), which confirms growth kinetics/morphological data.

**Fig. 5:**
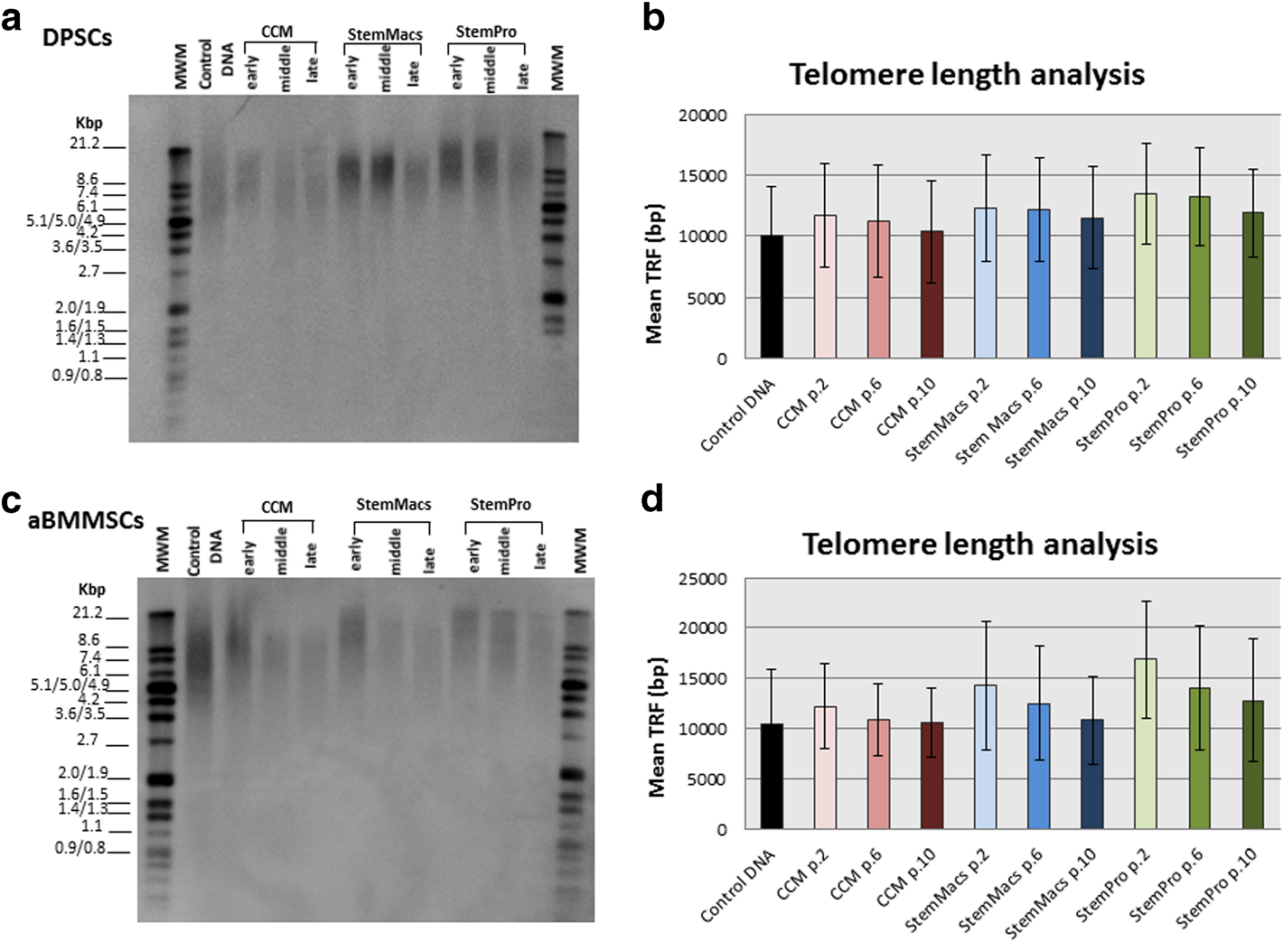
Impact of long-term expansion of DPSCs and aBMMSCs with three different culture media (CCM, StemMacs and StemPro) on telomere length. Telomere length determined by Southern blot analysis in cells harvested at early (p.2–3), middle (p.6–7) or late (p.10–11) passages. Lane 2 serves as a technical control, to confirm that the hybridization and Southern blot procedures were successful. Lanes 1 and 12 are molecular weight markers (kbp). **a, c** Representative Southern blots and **b, d** respective mean terminal restriction fragments (TRFs ± SD) for each medium/passage. A trend for lower mean TRF values could be observed with continuous passaging. This was consistent for all different culturing media and types of cells. Figures are representative of a single DPSC and aBMMSC donor for each culture medium. aBMMSC alveolar bone marrow mesenchymal stem cell, CCM complete culture medium, DPSC dental pulp stem cell, p. cell passage

To further evaluate the safety of oral MSC expansion, the telomere length was investigated as an additional measure of MSC aging [29] (Fig. 5a–d). A consistent decrease of telomere length was found in both DPSC and aBMMSC cultures under all culturing conditions. However, the noted telomere shortening was not statistically significant (*p* ≥ 0.2600) for any of the culture media or types of cells. Telomere length decreased from 11.6 (4.5) to 10.3 (4.2) kbp in CCM-expanded (*p* = 0.9740), from 12.2 (4.3) to 11.4 (4.2) kbp in StemMacs-expanded (*p* = 0.9825) and from 13.4 (4.1) to 11.8 (3.6) kbp in StemPro-expanded (*p* = 0.9329) DPSCs from p.2 to p.10; the respective values for the aBMMSCs were from 12.2 (4.5) to 10.6 (3.5) kbp in CCM-expanded (*p* = 0.9908), from 14.2 (6.3) to 10.8 (4.4) kbp in StemMacs-expanded (*p* = 0.9067) and from 16.8 (5.8) to 12.8 (6.1) kbp in StemPro-expanded (*p* = 0.9940) cells.

### Expression of lineage-specific markers

**Fig. 6:**
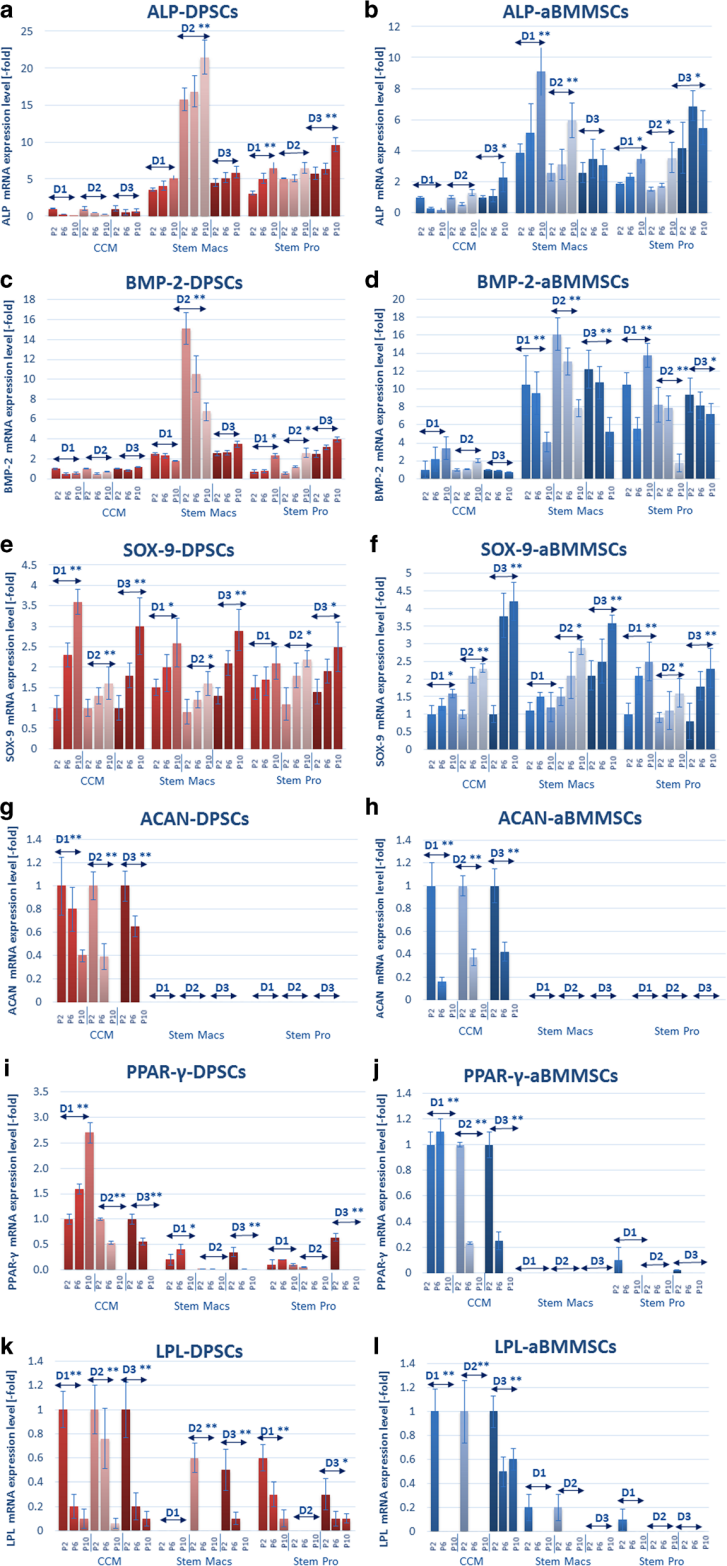
**a–l** Real-time PCR analysis of the expression of lineage-specific osteogenic (ALP, BMP-2), chondrogenic (SOX-9, ACAN) and adipogenic markers (PPAR-γ, LPL) in DPSCs (*n* = 3 donors, D1–D3) and aBMMSCs (*n* = 3 donors, D1–D3) after long-term expansion with three different culture media (CCM, StemMacs and StemPro). Values are mean (± SD) of three independent experiments (*n* = 3) in duplicate. Asterisks indicate statistically significant differences (*p < 0.05; **p < 0.01) in expression of each marker with passaging for each cell type/donor and medium separately. Comparisons between cells, passages and media were performed by two-way ANOVA followed by Tukey’s post-hoc tests. aBMMSC alveolar bone marrow mesenchymal stem cell, ACAN aggrecan, ALP alkaline phosphatase, BMP-2 bone morphogenetic protein, CCM complete culture medium, DPSC dental pulp stem cell, LPL lipoprotein lipase, P cell passage, D donor, PPAR-γ peroxisome proliferator-activated receptor gamma, SOX-9 sex-determining region Y-Box 9

Major differences were observed in lineage-specific gene expression patterns of oral MSCs expanded under serum-based vs serum-free conditions, demonstrating similar trends in both DPSCs and aBMMSCs, despite their differences in their origin. Specifically, both DPSCs and aBMMSCs expanded under serum-free conditions exhibited a significantly higher baseline (at early passages, p.2–3) expression of osteogenic markers (ALP, BMP-2), as compared to serum-expanded cells. Despite interindividual variations, this finding was consistent for most of the cell cultures, except for BMP-2 which was expressed in low levels at baseline by DPSCs expanded with StemPro, similarly to serum-expanded DPSCs. Further, the initially higher baseline expression of ALP in serum-free-expanded cells continued to increase with passaging (or occasionally remained stable), while the initially extremely high expression of BMP-2 significantly decreased (or remained stable in some StemMacs-expanded DPSC donors) (Fig. 6a–d). For StemPro-expanded DPSCs, the initially low BMP-2 expression significantly increased with passaging, unlike for CCM-expanded DPSCs where no significant upregulation of either ALP or BMP-2 generally occurred with prolonged expansion. It should be noted that all of these data were derived from simple passaging without the use of any osteogenic inductive medium.

Similar expression patterns of chondrogenic differentiation markers occurred in long-term expanded DPSCs and aBMMSCs. The chondrogenesis-related transcription factor SOX-9 was substantially upregulated in oral MSCs expanded under both serum-based and serum-free conditions in the majority of cell donors. In contrast, the baseline expression of ACAN gene, encoding the cartilage-specific proteoglycan core protein (CSPCP) or aggrecan, was downregulated in CCM-expanded oral MSCs and was entirely eliminated (no expression) in oral MSCs expanded with both serum-free media (Fig. 6e–h).

In contrast, the expression of PPAR-γ, an adipose tissue-located peroxisome proliferation-activated receptor, and of the adipose-tissue specific lipoprotein lipase (LPL) were in most cases downregulated (in serum-expanded oral MSCs, except PPAR-γ in donor 1) or completely eliminated (in serum-free expanded oral MSCs) after consecutive passaging (Fig. 6i–l).

Taken together, current data indicate that the prolonged expansion of oral MSCs under serum-free, cGMP conditions correlates with overexpression of osteogenesis-related markers, accompanied by complete elimination of adipogenesis and chondrogenesis-related markers, while serum-based expansion causes only minor such changes (i.e., relatively stable levels of osteogenesis-related genes, and moderate downregulation of chondrogenesis-related and adipogenesis-related genes, such as *ACAN* and *LPL*, respectively).

### Osteogenic and chondrogenic differentiation potential

To clarify whether the substantial differences in gene expression patterns during prolonged expansion also reflect differences of oral MSC differentiation potential, DPSCs and aBMMSCs at early, middle and late passages were induced to differentiate toward osteogenic and chondrogenic lineages. This allowed direct comparisons regarding the lineage-specific differentiation potential toward osteogenic and chondrogenic phenotypes (which are of paramount importance in TE) when different culture systems were used at various passages.

**Fig. 7:**
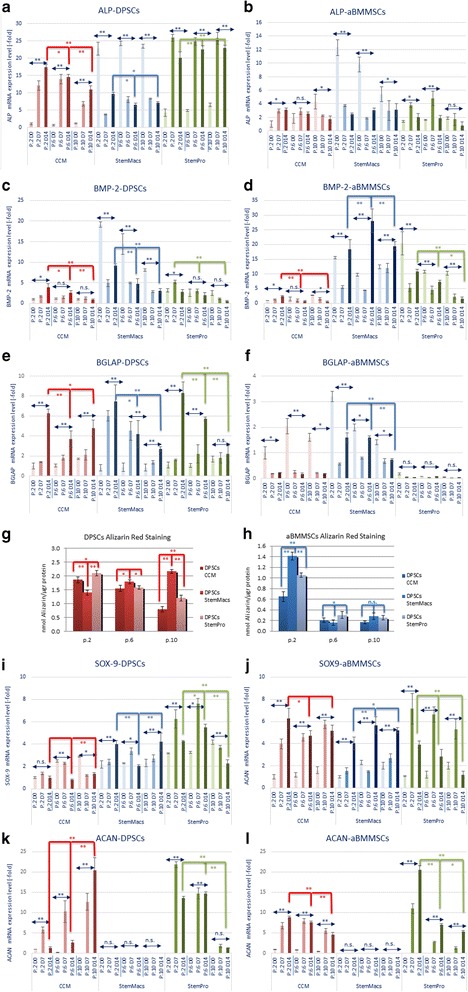
Real-time PCR analysis of the expression of osteogenic (ALP, BMP-2, BGLAP) and chondrogenic (SOX-9, ACAN) markers after induction of early, middle and late-passage DPSCs and aBMMSCs expanded previously with three different culture media (CCM, StemMacs and StemPro). Data are representative of one cell donor for each cell type and medium. Values are mean (± SD) of three independent experiments (*n* = 3) in duplicate. Asterisks over horizontal double arrows indicate statistically significant differences (*p < 0.05; **p < 0.01; n.s. = nonsignificant) at each passage of each cell type and medium, during the entire induction period (D0, D7 and D14). Asterisks over/under red, blue or green brackets indicate statistically significant differences (*p < 0.05; **p < 0.01) in the expression of each marker among different passages (p.2 vs p.6 vs p.10) at D14 after induction of differentiation. **a–f** Expression of osteogenic markers (ALP, BMP-2, BGLAP). **g**, **h** Spectrophotometric quantification of the Alizarin Red S staining of DPSCs and aBMMSCs induced for osteogenic differentiation after 21 days (mean nmol AR-S/μg of total protein ± SD of three independent experiments of a representative donor in three replicates). **i–l** Expression of chondrogenic markers (SOX-9, ACAN) at D0, D7 and D14 after induction. Comparisons between cells, passages and media performed by two-way ANOVA followed by Tukey’s post-hoc tests. aBMMSC alveolar bone marrow mesenchymal stem cell, ACAN aggrecan, ALP alkaline phosphatase, BGLAP bone gamma-carboxyglutamate protein, BMP-2 bone morphogenetic protein, CCM complete culture medium, DPSC dental pulp stem cell, P cell passage, D day, SOX-9 sex-determining region Y-Box 9

Serum-based medium (CCM)-expanded DPSCs sustained the potential for osteogenic differentiation from early to late passages, indicated by significant, time-dependent upregulation of ALP (*p* < 0.0001 for all passages), BMP-2 (only for early and middle passages, *p* < 0.0001) and BGLAP (*p* < 0.0001 for all passages) up to day 14 following induction. This potential was significantly higher in early-passaged cells but gradually diminished in middle and late-passaged cells, evidenced by the comparative expression of ALP, BMP-2 and BGLAP at day 14 post induction (Fig. 7a, c, e). In contrast, CCM-expanded aBMMSCs enabled upregulation of ALP (*p* = 0.0098) and BMP-2 (*p* = 0.0123) (but not BGLAP that was actually significantly downregulated) only at early passages, while this ability diminished at middle and late passages (Fig. 7b, d, f).

StemMacs-expanded DPSCs showed significant downregulation of the extremely high expression of ALP (*p* < 0.0001 for all passages) and BMP-2 (*p* < 0.0001 for early and middle; *p* = 0.0031 for late passages) present at baseline (as described in previous paragraph and Fig. 6), while the initially low expression of the late mineralization marker BGLAP gradually increased (*p* < 0.0001 for early; *p* = 0.0075 for middle; nonsignificant for late passages, *p* = 0.1150) up to 14 days (Fig. 7a, c, e). The ALP and BMP-2 downregulation was subdued in early-passaged compared to middle and late-passaged cells, while the BGLAP upregulation was more pronounced at early compared to late passages.

In StemMacs-expanded aBMMSCs the initial extremely high expression of ALP (*p* < 0.001 for all passages, except late *p* = 0.0717) and BGLAP (*p* < 0.0001 for all passages) declined (Fig. 7b, f), in contrast to BMP-2 which demonstrated a continuously increasing expression, reaching a peak at the middle passages (Fig. 7d). Multiple comparisons between early, middle and late-passage groups showed that BMP-2 upregulation was significantly higher for middle passages (*p* = 0.0003 compared to early passages at day 14), while BGLAP downregulation was less pronounced at early compared to middle and late passages.

Regarding the StemPro-expanded DPSCs, a significant upregulation of ALP (*p* < 0.0001 for all passages) as well as of BMP-2 and BGLAP expression was found during osteogenic differentiation (*p* < 0.0001 for both markers but only at early and/or middle passages) (Fig. 7a, c, e). Multiple comparisons showed that there were no major differences in ALP expression among passages, while BMP-2 and BGLAP expression was more pronounced at early and/or middle passages (Fig. 7a, c, e). Finally, in StemPro-expanded aBMMSCs ALP was upregulated only at early (*p* = 0.0059) and middle (*p* = 0.0009) passages, whereas BMP-2 and BGLAP were downregulated or remained stable respectively (Fig. 7b, d, f).

In line with these data, the AR-S-based mineralization assay confirmed that DPSCs expanded with all three-media retained a gradually declining (for CCM and Stem Pro; *p* < 0.0001) or increasing (for StemMacs; *p* < 0.0001) mineralization potential after prolonged passaging. However, aBMMSCs showed a higher mineralization potential when expanded under serum-free as compared to serum-based conditions at early passages (*p* < 0.0001 for comparisons of CCM with both StemMacs and StemPro), while this potential was entirely eliminated for all media at middle and late passages.

A similar analysis for chondrogenic differentiation potential pointed out that CCM-expanded DPSCs demonstrated an increasing chondrogenic differentiation potential with passaging, as signified by increasing expression of ACAN (*p* = 0.0324, *p* = 0.0003 and *p* < 0.0001 at early, middle and late passages, respectively, at day 7 post induction); these effects were more pronounced at late as compared to middle and early passages (Fig. 7i, k). In contrast, CCM-expanded aBMMSCs showed a significant gradual decline in chondrogenic differentiation potential with passaging, as evidenced by diminishing upregulation of ACAN (Fig. 7j, l). For the two remaining media, it was shown that StemMacs-expanded DPSCs and aBMMSCs notably lost their entire chondrogenic potential at all passages, while StemPro-expanded DPSCs and aBMMSCs showed a remaining chondrogenic potential, indicated by the expression of SOX-9 and ACAN that, however, declined with passaging (Fig. 7i–l).

## Discussion

The discovery of the unique biological properties of human MSCs has generated a plethora of clinical trials designed to test their safety and efficacy for the treatment of various pathologies. In dental research, a number of case reports have been conducted to test the regenerative potential of oral MSCs in orofacial osseous [30] and pulp regeneration [31], whereas randomized controlled clinical trials (RCTs) are quite sparse especially in humans [32, 33]. An electronic search in the ClinicalTrials.gov database (accessed July 2017) implemented by the US National Institutes of Health revealed six ongoing clinical trials administering oral MSCs in various applications, including periodontal and peri-implant therapies, cleft lip and palate management and revitalization of immature permanent teeth with necrotic pulp [34]. Nevertheless, the clinical application of MSC-based therapies has been constrained, among other reasons, by difficulties in the ex-vivo expansion of clinical-grade MSCs under good manufacturing practice (GMP) conditions, in compliance with EU regulations [6, 8]. To our knowledge, this is the first comprehensive study providing extensive characterization of oral MSCs (DPSCs and aBMMSCs) after prolonged expansion with two proprietary culture systems fulfilling cGMP requirements, as compared with a conventional serum-based medium.

It is widely accepted that differing culture conditions may affect MSC properties [3]. Since preparation of high-quality MSCs is required for any safe and efficient cell therapy treatment, considerable efforts have been made to evaluate the consequences of cultivation processes on stem cell behavior. It has been reported that prolonged culture of bone marrow stromal cells correlates with various functional changes, including reduced proliferation, ultimately producing replicative senescence [9] and associated molecular changes [35], together with reduced [36] or shifting multilineage differentiation potential from highly osteogenic to more adipogenic [37]. However, currently there is not much in-depth knowledge regarding the precise effects of the culture micro-milieu on the “stemness” properties of oral MSCs. The present study has performed a rigorous parallel investigation of two oral MSC populations of different origin (i.e., from adult dental pulp (DPSCs) and alveolar orofacial bones (aBMMSCs)), both recommended for dental tissue and orofacial bone TE, respectively [14, 17, 30, 31]. The extensively used animal serum (bovine or calf) and porcine-derived dissociation agent trypsin, although still tolerated by the regulatory authorities as being used in ongoing clinical trials, are not considered compatible with cGMP practices, while other serum-based cGMP alternatives, including human autologous or allogeneic serum [38] or HPL [39], impose several limitations regarding large-scale cell expansion [40] and reagent availability. Based on these shortcomings, this study sought to trace potential quenching of critical stem cell properties after isolation and subsequent prolonged expansion of oral MSCs under serum/xeno-free, cGMP compared to conventional serum-based culturing conditions.

There is a growing effort by leading life science companies to develop serum/xeno-free media formulations that are in full compliance with GMP requirements, as defined by the EU, in addition to relevant “supportive” reagents, namely cGMP culture vessel coatings, dissociation and cryopreservation reagents, and so forth, in order to fully support clinical-grade production of MSCs for therapeutic use. However, a basic drawback is that the manufacturers do not provide any information on the chemical formulations of these proprietary media, other than certificates on their GMP compliance. On the other hand, there are quite a large number of developed serum-free stem cell expansion media provided by several companies, mainly aiming to replace calf/bovine serum due to its well-known disadvantages. These media are characterized as serum free, but not as cGMP.

There is quite rigorous and well-performed, previously published work on how the chemistry of various “house-made” serum-free media may affect “stemness” properties, such as PDs and phenotype expression of the stem cells. More specifically, several authors have investigated the effects of a wide range of medium supplements (including FGF-2, PDGF-BB, ascorbic acid, EGF) as well as other culture medium ingredients (such as glucose content, addition of l-glutamine vs Glutamax) on “stemness” properties of various MSC types, with variable results [11, 41–45]. However, very often there is a lack of direct comparison between paired samples, thus making it difficult to outweigh the actual effects of switching supplements. Furthermore, these disclosed formulations should ensure the biosafety of the media ingredients, which requires further testing.

In order to address these challenges by “in-house” serum-free media of known consistencies, newly developed proprietary serum/xeno-free, cGMP media have been developed as promising alternatives for the manufacturing of clinical-grade MSCs. Until now, there are sparse comparative studies evaluating these proprietary cGMP media and their impact on “stemness” properties of various types of MSCs [46–49]. Nevertheless, in most of these studies, StemPro (Life Technologies) is evaluated and proposed as a promising alternative to classic serum-containing media. For this reason, the current study elected to use this medium and make direct comparisons of two different oral stem cell populations to other nonoral MSC types. StemMacs, on the other hand, belongs to a new generation of recently developed cGMP culture systems and therefore was also included in this study, since comparisons of more than one serum/xeno-free, cGMP medium with a standard (FBS) serum-based medium documents a more comprehensive characterization of the innate potential of the oral MSCs to support the initial isolation procedures, and their robustness to provide adequate cell numbers over prolonged serum-free expansion.

Research findings of the present study demonstrated similar isolation efficiency under serum-free conditions for both DPSCs and aBMMSCs, as the required time to reach p.1 for each cell type was similar for the three media tested, although the overall time was higher for aBMMSC (approximately 12–14 days) compared to DPSC (approximately 9–11 days) cultures. This could be partially attributed—except from the obvious reason of different tissues of origin—to the younger age of the DPSC donors over the aBMMSC donors, since age is known to significantly affect biological properties such as the proliferation rate [50] and to be associated with epigenetic alterations, telomere attrition, accelerated cellular senescence, stem cell exhaustion and altered intercellular communication [51]. In addition, the expanded DPSCs under serum-free conditions produced similar growth patterns during consecutive passaging compared to the serum-based conditions (Fig. 1), whereas StemMacs-expanded aBMMSCs showed a pronounced increase in PDT after middle passages (p.6–7). However, it has to be mentioned that all media were able to achieve cell yields equal to (aBMMSCs) or greater than (DPSCs) 30 million cells—corresponding to a generally recommended clinical dose for intraoral applications—after completion of no more than three (early) passages. Several commercial serum/xeno-free media have recently emerged in the market as promising tools for establishing clinical-grade MSCs with enhanced “stemness” characteristics. A few studies have investigated various serum-free proprietary media as promising alternatives to in-house-developed serum-free media [46–49] and have shown that these products are able to isolate and expand various MSC populations, adhering to the minimal criteria set by the International Society of Cellular Therapy (ISCT) and often providing an enhanced performance compared with the conventional media [52]. However, some of these studies are not standardized for several parameters, such as proliferation rates or variability in the differentiation potential of the cells among media. The present study highlighted the importance of thoroughly exploring MSC properties of different cell populations grown in various serum-free media, as the highly proliferative DPSCs were not severely inhibited—at least regarding cell growth—under both serum-free media, while the less proliferative aBMMSCs showed a dramatic elimination of proliferation dynamics after prolonged expansion with one of these media (StemMacs). As new generations of serum-free media emerge from various companies, these data may be significant in addressing particular challenges related to the preparation of oral MSCs for therapeutic use.

Notably, growth kinetics were not fully accordant with morphological differences and senescence data, implying that multiple criteria have to be considered to fully characterize “stemness” of oral MSCs. DPSCs expanded under serum-free conditions showed similar growth patterns to serum-expanded cells (Fig. 1), but exhibited increased SA-β-gal positivity (especially at late passages) (Fig. 4), accompanied by a generally elevated cell size distribution and granularity (Fig. 2). In contrast, aBMMSCs contained significantly more SA-β-gal-positive cells compared to DPSC cultures, which was more pronounced in the StemMacs-expanded cells, in accordance with the growth and morphological data (Figs. 1, 2 and 4). The limitation of cell division in cultures is a known phenomenon for all somatic cells unless the cells are immortalized [53]; after approximately 40–100 divisions, depending on cell type and culture conditions, the proliferation rates decay, with eventual domination by the senescent state [46]. Nevertheless, it should be noted that, despite increased numbers of SA-β-gal-positive cells, the reduction in the mean telomere length with increasing passage number, although evident, remained minor, failing to reach statistical significance for all of the cells/media studied (Fig. 5). Telomere length is a primary indicator of MSC replicative (telomere-associated) senescence [29, 54] characterized by progressive loss of the telomeric TTAGGG repeats [55]. The results of this study suggest that variations in growth kinetics among different oral MSC types and/or expansion media are not directly attributable to accelerated telomere loss. It has been contended that SA-β-gal activity, although associated, is neither causative nor specific for senescence [54, 56, 57]. Instead, it should be interpreted in combination with other biomarkers [56, 58]. However, given the expression of β-galactosidase has been mostly associated with a nonproliferative state, the increased proportion of such cells may be implicated in the disparate growth characteristics observed for different oral MSCs and/or expansion systems. In line with present findings, a previous study [59] showed no significant shortening (<1 kbp) in mean telomere length of aBMMSCs at early (p.1–4; PD < 25) and middle (p.5–8; PD < 50) passages, while at the same time significantly greater proportions of SA-β-gal-positive cells became evident over time. It has also been shown that significant heterogeneity existed among different DPSC subpopulations, with those capable of exceeding 80 PDs before senescence occurred possessing longer telomeres (18.9 kbp) than the less proliferative populations (<40 PDs) which had much shorter telomeres (5–13 kbp) [60]. Although there is a general recommendation that expansion of bone marrow MSC preparations for clinical use should be limited to 4–7 PDs [61], the current study, in addition to previous work, aimed to look into the replicative potential of these MSC populations and set the limits and standards for effective cell expansion in order to attain the desired cell numbers before senescence occurs. Indeed, activation of senescence has been demonstrated to produce morphologic and functional changes such as alterations in nuclear structure, protein processing, gene expression and cell metabolism [62] that might compromise the clinical outcome. In addition, senescence has been associated with the so-called “senescence-associated secretory phenotype” (SASP), which might be detrimental to regeneration as the senescent cells may release numerous cytokines, growth factors, proteases and extracellular matrix components inducing a state of chronic inflammation [63]. It has been further shown that decreased expression of cytokine and chemokine receptors in aged BMMSC populations compromises their protective role by limiting potential for activation and mobilization to the injury site [64].

Of interest was that oral MSCs expanded with serum-free media exhibited a very homogeneous morphology comprising well-aligned spindle-shaped cells (Fig. 2). This finding is in accordance with previous data [65] and also with the specifications of the manufacturers regarding the selective isolation of homogeneous MSC populations. However, knowing the exact composition of these media is essential to justify such postulations. In contrast, serum-expanded oral MSCs (both DPSCs and aBMMSCs) showed higher population heterogeneity comprising cells of various shapes and sizes. Heterogeneity of the cells isolated by the enzymatic dissociation method that are “loosely” termed “MSCs” [66] has been related to cultures containing cells at different stages of developmental commitment, including stem cells, rapidly dividing transit amplifying (TA) cells that become progressively lineage restricted while dividing [60].

The present study illustrated the variable expression patterns of MSC markers as important hallmarks of “stemness”. Well-recognized MSC markers such as CD90 and CD73 in addition to CD81 and CD49f were stably and abundantly expressed in both cell types under serum-based as well as serum-free conditions up to late passages (Fig. 3a–f), thus being totally unaffected by any of the observed morphological, growth, senescence or differentiation changes of the cells. In contrast, other MSC markers such as CD105, CD146, STRO-1 and the embryonic markers SSEA-1 and SSEA-4 were more sensitive in “following” such morphological/biochemical alterations after prolonged passaging, and showed more pronounced downregulation during serum-free-based (all markers except SSEA-1 reduced at similar levels) as compared to serum-based expansion of both cell types (Fig. 3a–f; Additional file 1). These findings contrast with a recent study [49] that demonstrated a very low and unaltered expression of CD105 and SSEA-4 with prolonged passaging in human DPSCs, while other studies confirmed downregulation of SSEA-4 during differentiation in human MSCs [59]. SSEA-4 has been characterized as a classical embryonic stem cell marker used in isolating adult MSCs with enhanced multilineage differentiation potential [67] and capacity for forming organized bone tissue in vivo [68].

The present study also confirmed the upregulation of the initially minimally expressed CD34 (<10%) after prolonged expansion; a consistently apparent donor-related finding both in DPSC and aBMMSC cultures irrespective of the culture conditions. A recent concise review [69] reports that although CD34 is associated with the selection of hematopoietic stem cells from bone marrow transplants, it is also expressed by MSCs, as well as by other nonhematopoietic cell types, indicating a distinct cell subset with enhanced progenitor activity. Therefore, the ISCT consensus of CD34 as a negative MSC marker has not been verified by some reports, which demonstrate that CD34 is an important marker in early, freshly isolated MSCs, associated with high colony forming efficiency and prolonged proliferative capacity, but whose expression gradually diminishes with passaging [70]. Current results showed increasing CD34 expression with passaging, in line with a previous report [70] which found an increase of baseline expression of CD34 in adipose MSCs at 15 days followed by downregulation at 30 days, while CD90 remained consistently highly expressed, as in the present study. Increased CD34 expression was related to the acquirement of proendothelial characteristics, since CD34 was also highly expressed in endothelial progenitors [71]. Investigation of the angiogenic potential of oral MSCs after prolonged culture could clarify this position.

Present results also highlighted that expansion with both serum-free systems resulted, even at very early passages (p.2–3), in a “predisposition” toward an increased baseline expression of osteogenic markers, with parallel complete elimination of chondrogenic (the late marker ACAN, counteracted by overexpression of the transcription factor SOX-9) and adipogenic markers (both the early and late markers PPAR-γ and LPL, respectively), whereas serum-based expansion comparatively produced only minor changes in osteogenic markers and less prominent changes in adipogenic and chondrogenic markers (Fig. 6a–l). Evidence indicates that osteogenesis and adipogenesis are inversely regulated by their master transcription factors, Runx2 and PPAR-γ, respectively [72]. Chondrogenesis and osteogenesis share common transcriptional regulatory pathways via activation of Runx2/Cbfa1, but deviate in several other control points. In particular, chondrogenesis is strongly regulated by SOX-9 that remains continuously present from early stages of MSC condensation to become chondrocytes until late stages of chondrocyte hypertrophy [72]. SOX-9 regulates expression of cartilage extracellular matrix proteins, such as Col2A1 and ACAN [73]. It is therefore remarkable that SOX-9 upregulation was accompanied by elimination of expression of ACAN. A possible explanation could be that this effect is due to a negative transcription regulation, and warrants further investigation.

We further evaluated the impact of this “osteogenic predisposition” of serum-free expanded oral MSCs on their multilineage differentiation potential toward osteogenic and chondrogenic phenotypes. Notably, serum-expanded DPSCs retained a diminishing osteogenic differentiation potential with passaging, while serum-expanded aBMMSCs lost this potential by reaching the middle passages (Fig. 7a–f). This finding concurs with previous reports of reduced multipotency of DPSCs [74] and BMMSCs from other sources [15, 75], and is also consistent with the mineralization potential analysis data (Fig. 7g, h). On the other hand, StemMacs-expanded DPSCs and aBMMSCs showed a diminishing expression of most initially highly expressed osteogenic markers during osteogenic differentiation. However, the mineralization potential increased in DPSCs with passaging, and in the case of aBMMSCs the initially very high mineralization potential was eliminated after reaching middle passages. The conclusion that can be drawn for this part is that the overexpression (compared to the serum-based conventional conditions) of osteogenic markers in StemMacs-expanded DPSCs is related to an increasing in-vitro mineralization potential with passaging, while in the case of aBMMSCs this advantage over serum-based expansion could be only observed at early passages. Finally, StemPro-expanded DPSCs showed further increase of most of the initially highly expressed osteogenic markers (at least at early to middle passages), which was also partially evident (only for ALP) in the StemPro-expanded aBMMSCs. This increase was attenuated with passaging, however, and this was also true for the in-vitro mineralization potential of both cell types, which decreased for the DPSCs and was eliminated for the aBMMSCs. It is therefore obvious that the overexpression of osteogenic markers in StemPro-expanded DPSCs and aBMMSCs is not related to a sustainable in-vitro mineralization potential with passaging, as compared to the serum-based conventional conditions. Overall these results highlight the need for carefully selecting cell populations and media to meet the needs of a particular clinical situation, taking into account that these cells can be actually “trusted” to maintain the highest degree of osteogenic potential only at early passages; the latter statement referring to both serum-based and serum-free conditions.

Finally, chondrogenic marker expression increased with passaging in serum-expanded DPSCs but decreased in serum-expanded aBMMSCs. Serum-free expansion was associated with a dramatic decrease (in StemPro-expanded DPSCs and aBMMSCs) or a complete elimination (in StemMacs-expanded cells) of the baseline chondrogenic marker expression, further confirming that there seems to exist a “selection” of the serum-free-expanded populations toward osteogenic progenitors. These data, however, require further functional confirmatory assays before incorporating them into standardized protocols.

## Conclusions

The present study provides novel insights into the critical parameters that define oral MSC “stemness” mostly influenced by the culture “micro-milieu” and have the potential to predict the isolation efficiency, growth and functional properties of MSCs before clinical application. It is evident that several biological endpoints, including morphological, growth, senescence, immunophenotypic, gene expression and differentiation characteristics, have to be comprehensively considered to assess maintenance of oral MSC “stemness”. It also becomes apparent that the establishment of new protocols of clinical-grade, cGMP expansion of MSCs is not trivial, and the feasibility of cGMP production of MSC preparations that meet the regulatory criteria is often ambivalent. Other parameters, such as the replacement of recombinant enzymes (collagenase and dispase) with cGMP reagents (e.g., the recently proposed highly purified GMP-grade enzyme blend named liberase; Roche Custom Biotech, Mannheim, Germany) or even complete restraint for enzymes (i.e., using the explant culture method [25]), also require consideration. The results of this study clearly show the feasibility of isolating and expanding DPSCs and aBMMSCs using cGMP, serum/xeno-free culture systems without compromising basic “stemness” properties at least in early (for aBMMSCs) or up to middle (for DPSCs) passages. The high baseline expression of osteogenic markers in serum-free-expanded MSCs could be of advantage in bone and/or other mineralized tissue (e.g., dentin) regeneration applications. Finally, typical MSC markers such as CD90 and CD73 appear not to be specific in indicating maintenance of “stemness” and should therefore be supplemented by multiparametric immunophenotyping, including at least CD105, CD146, SSEA-1, SSEA-4 or other embryonic SC markers. Specific additional challenges, such as the extremely high cost of these new cGMP media, also need to be taken into consideration for large-scale clinical applications. Finally, as different media produce MSCs with differential “stemness” properties, the most suitable protocol for clinical-grade MSC expansion should be carefully selected and validated in preclinical animal models to fit the regenerative requirements of the target tissues.

## Additional file

Additional file 1: is representative flow cytometry diagrams showing alterations in MSC marker expression in DPSCs during long-term expansion with CCM, StemMacs and StemPro (green line, unstained control; red line, marker of interest). Similar trends were observed for all DPSC and aBMMSC donors (PDF 418 kb)

## Abbreviations

aBMMSC: Alveolar bone marrow mesenchymal stem cell
ACAN: Aggrecan or cartilage-specific proteoglycan core protein (CSPCP)
ALP: Alkaline phosphatase
APC: Allophycocyanin
ATMP: Advanced therapy medicinal product
B2M: Beta-2-microglobulin
BGLAP: Bone gamma-carboxyglutamate protein or osteocalcin
BMP-2: Bone morphogenetic protein 2
CBMP: Cell-based medicinal product
CCM: Complete culture medium
CD: Cluster of differentiation
cGMP: Good manufacturing practice compliant
DPSC: Dental pulp stem cell
ECL: Enhanced chemiluminescence
EDTA: Ethylene-diamine-tetraacetic acid
EU: European Commission
FBS: Fetal bovine serum
FITC: Fluorescein isothiocyanate
FSC: Forward scatter
gDNA: Genomic DNA
HPL: Human platelet lysate
ISCT: International Society of Cellular Therapy
LPL: Lipoprotein lipase
MSC: Mesenchymal stem cell
OD: Optical density
p: Passage
PCR: Polymerase chain reaction
PD: Population doubling
PDT: Population doubling time
PE: Phycoerythrin
PerCP-Cy5.5: Peridinin-chlorophyll-protein-cyanin 5.5
PPAR-γ: Peroxisome proliferator-activated receptor gamma
SASP: Senescence-associated secretory phenotype
SA-β-gal: beta-galactosidase
SD: Standard deviation
SDHA: Succinate dehydrogenase complex, subunit A, flavoprotein
SFM: Serum-free medium
SOP: Standard operating procedure
SOX-9: Sex-determining region Y-Box 9
SSC: Side scatter
SSEA: Stage-specific embryonic antigen
TA: Transit amplifying
TE: Tissue engineering
TRF: Terminal restriction fragment
UNG: Uracil-*N*-glycosylase
