# Supplementary material for: Isolation and prolonged expansion of oral mesenchymal stem cells under clinical-grade, GMP-compliant conditions differentially affects “stemness” properties
Source: Stem Cell Res Ther. 2017 Nov 2;8:247. doi: 10.1186/s13287-017-0705-0 (PMC5667471; doi:10.1186/s13287-017-0705-0)
Supplement: Additional file 1: — is representative flow cytometry diagrams showing alterations in MSC marker expression in DPSCs during long-term expansion with CCM, StemMacs and StemPro (green line, unstained control; red line, marker of interest). Similar trends were observed for all DPSC and aBMMSC donors (PDF 418 kb) [file 13287_2017_705_MOESM1_ESM.pdf]

CD73

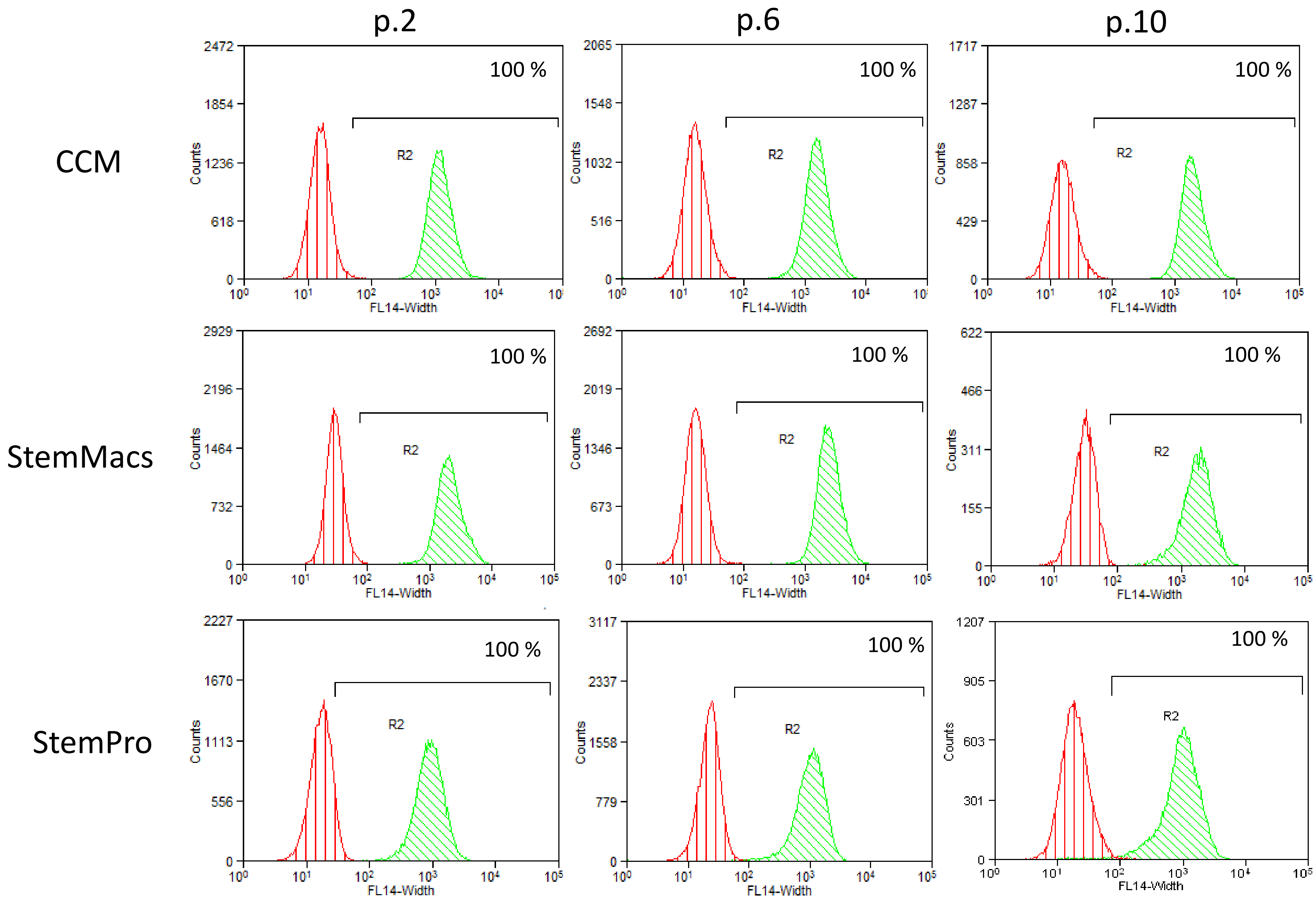

CD90

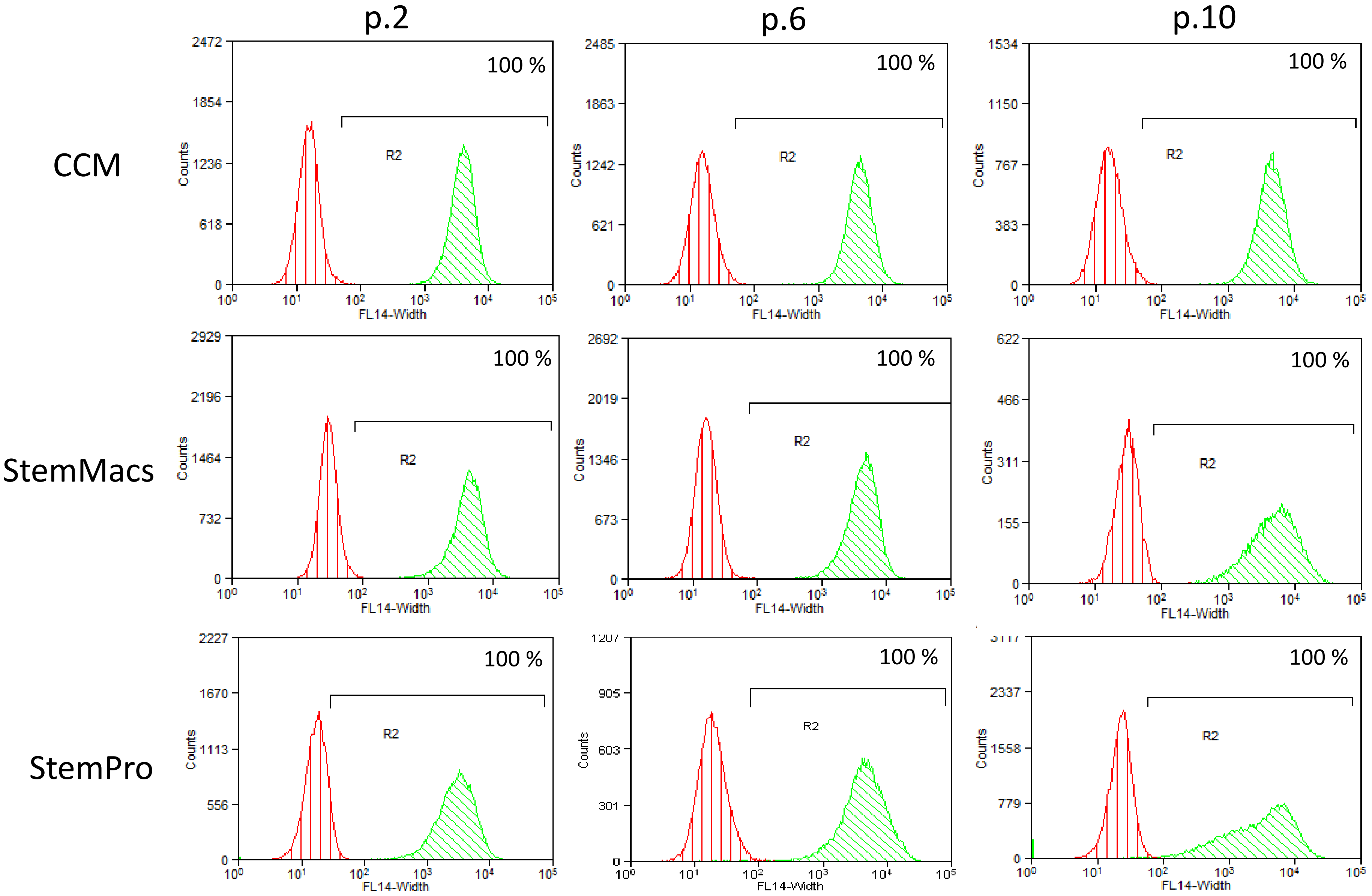

CD81

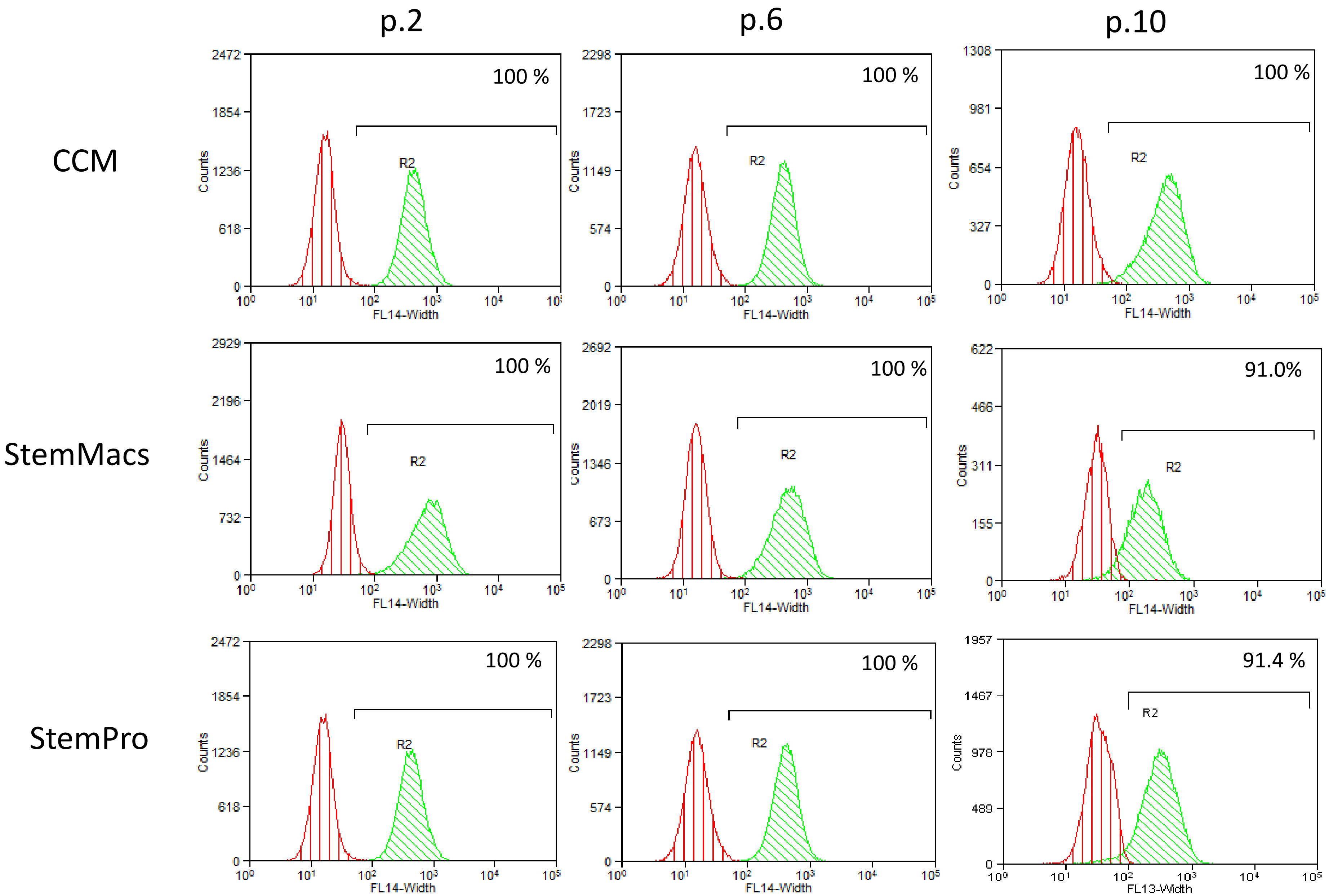
p.2
p.6
p.10
CCM
StemMacs
StemPro
100 %
100 %
100 %
100 %
100 %
91.0%
100 %
100 %
91.4 %
R2
Counts
FL14-Width
FL13-Width

CD49f

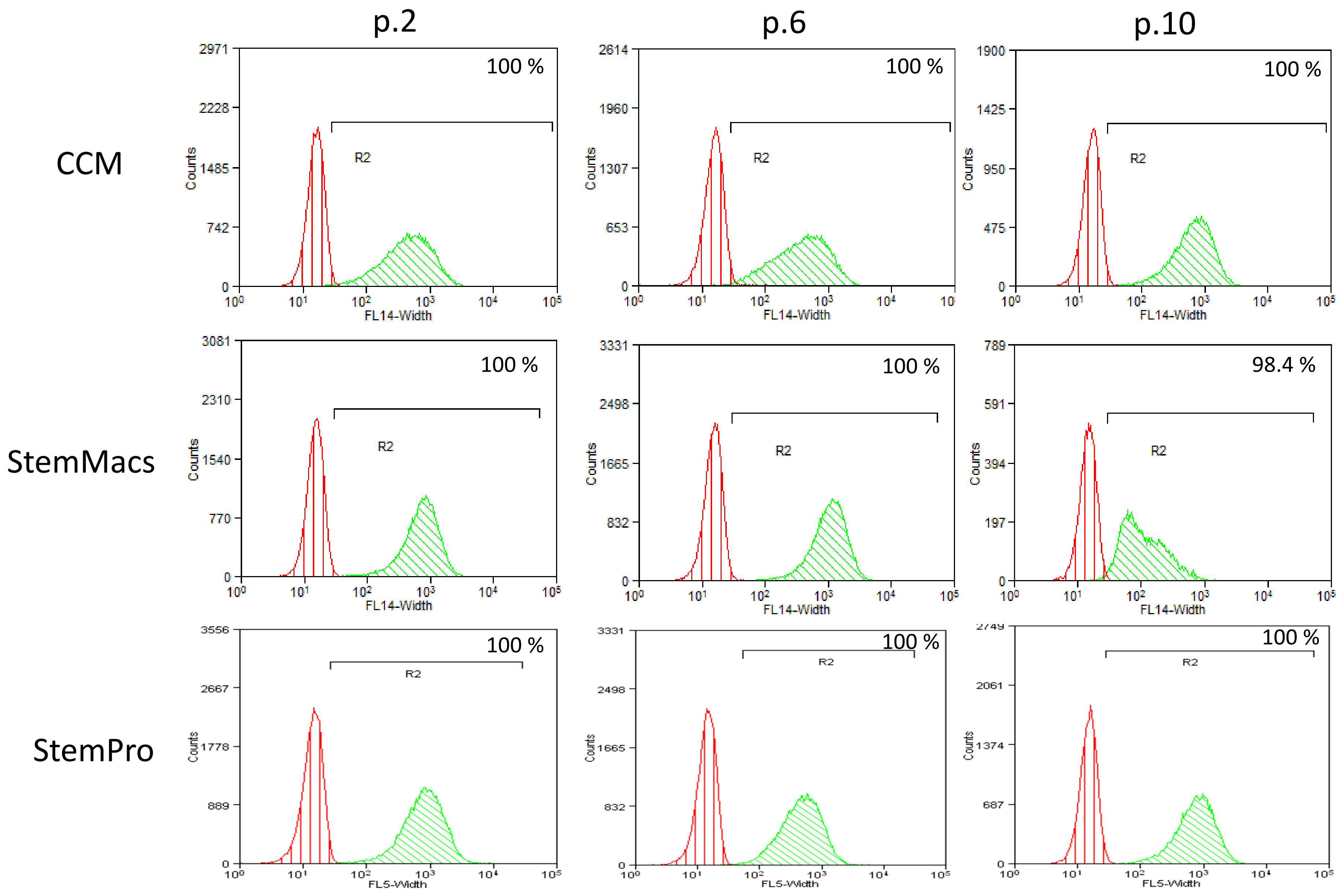

p.2
p.6
p.10
CCM
StemMacs
StemPro
100 %
100 %
100 %
100 %
100 %
98.4 %
100 %
100 %
100 %
R2
Counts
FL14-Width
FL5-Width

CD146

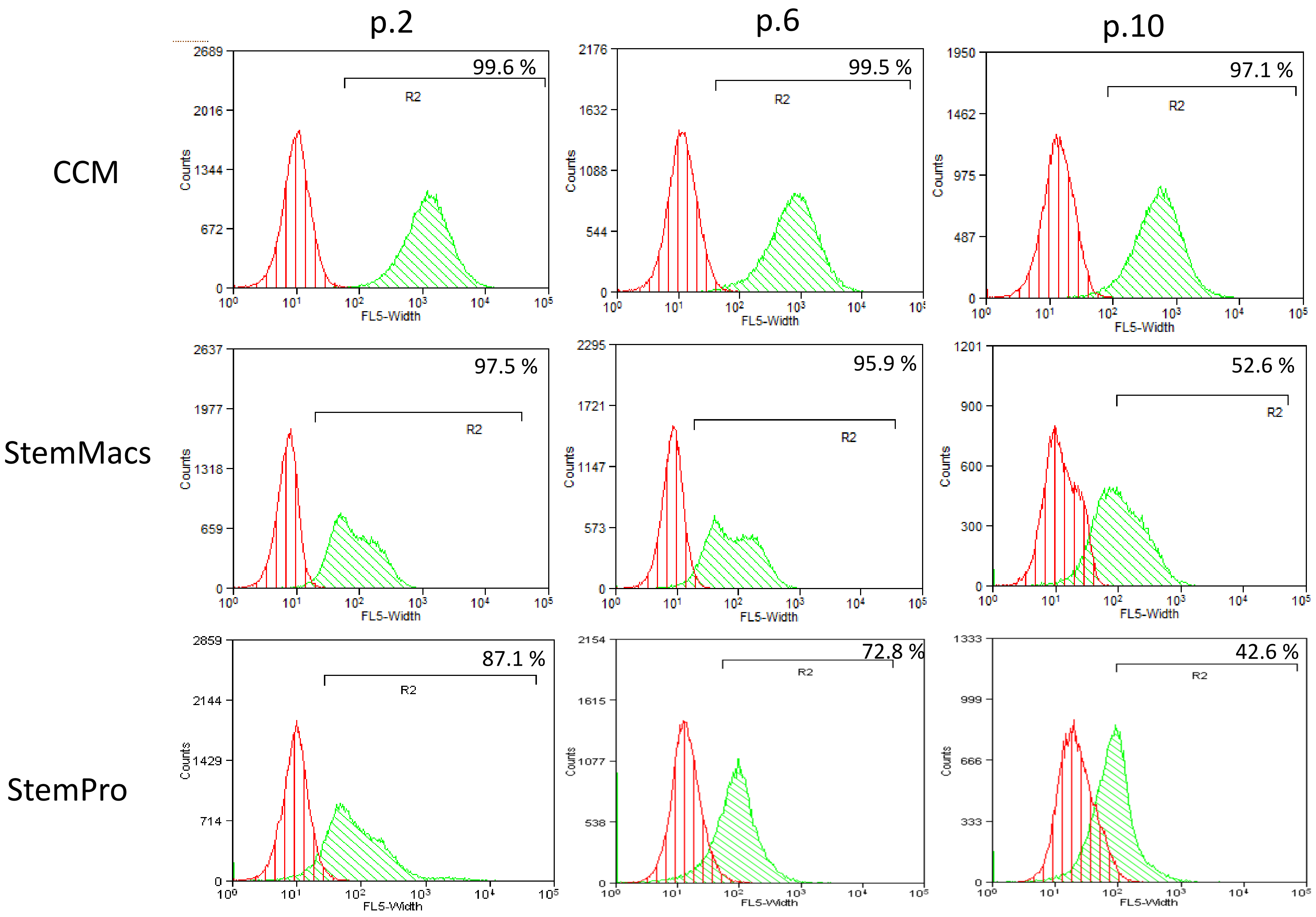

p.2
p.6
p.10
CCM
StemMacs
StemPro
99.6 %
99.5 %
97.1 %
97.5 %
95.9 %
52.6 %
87.1 %
72.8 %
42.6 %
R2
Counts
FL5-Width

CD105

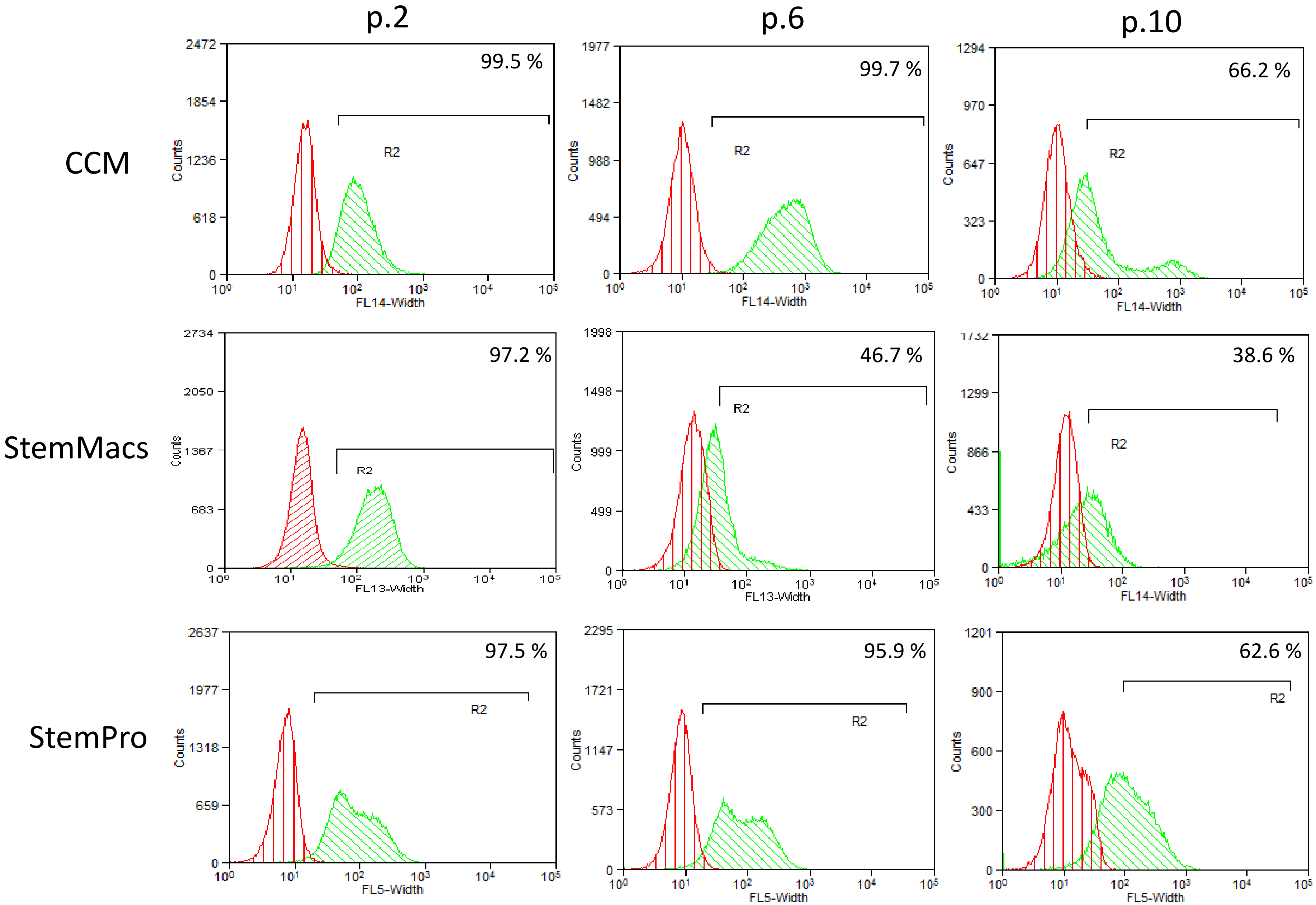

p.2
p.6
p.10
CCM
StemMacs
StemPro
99.5 %
99.7 %
66.2 %
97.2 %
46.7 %
38.6 %
97.5 %
95.9 %
62.6 %
R2
Counts
FL14-Width
FL13-Width
FL5-Width

STRO-1

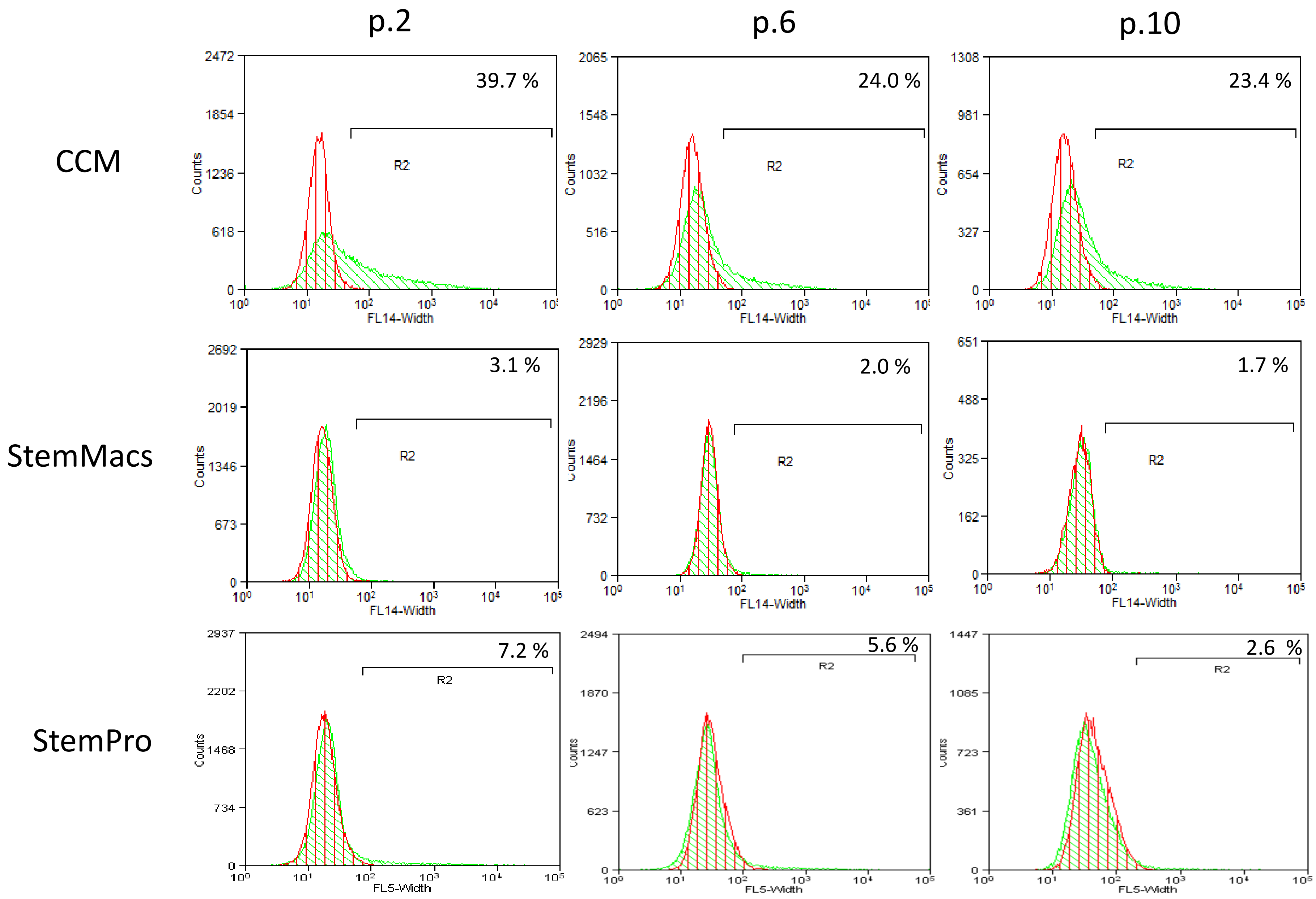

p.2
p.6
p.10
CCM
StemMacs
StemPro
39.7 %
24.0 %
23.4 %
3.1 %
2.0 %
1.7 %
7.2 %
5.6 %
2.6 %
R2
Counts
FL14-Width
FL5-Width

SSEA-4

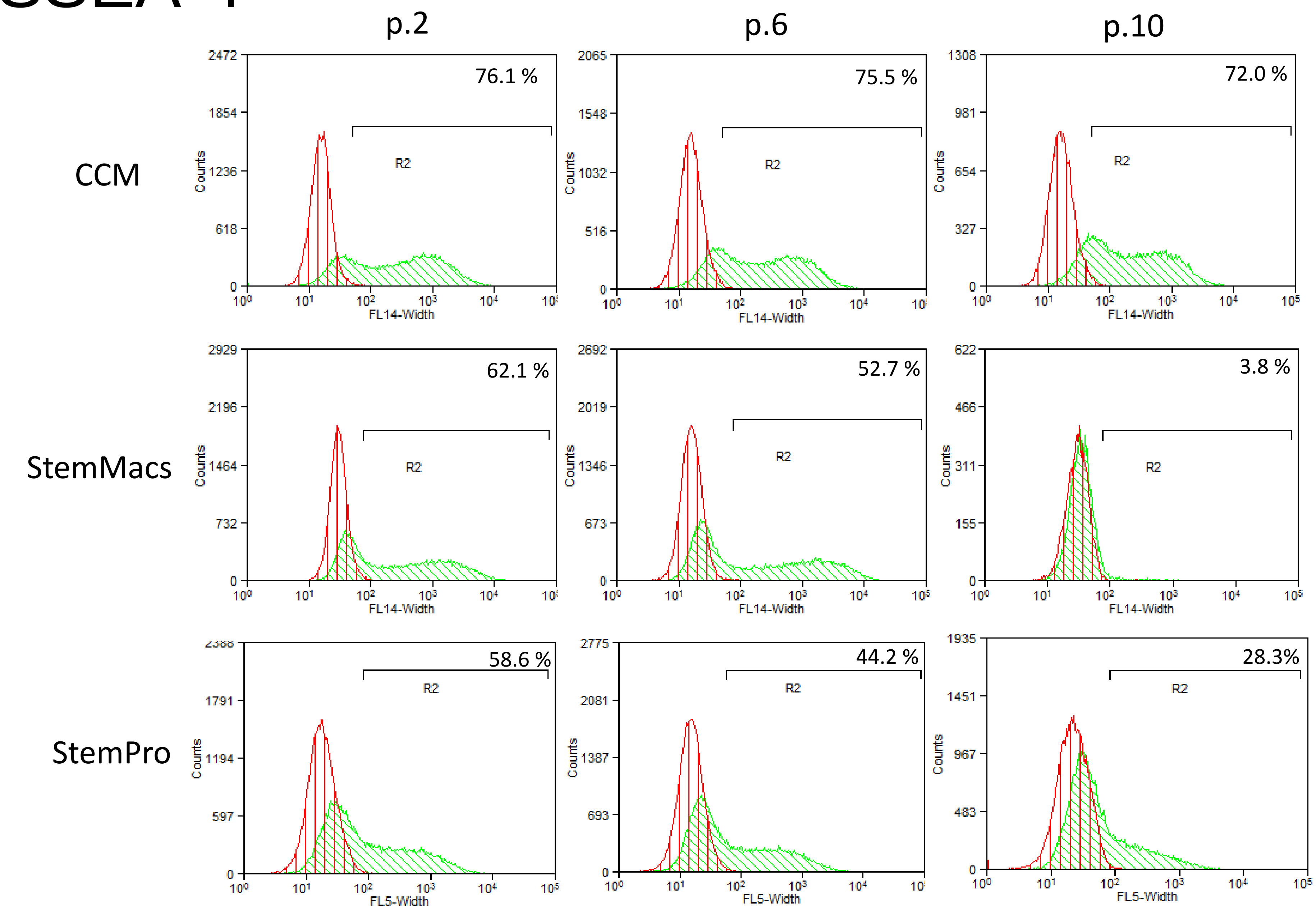
p.2
p.6
p.10
CCM
StemMacs
StemPro
76.1 %
75.5 %
72.0 %
62.1 %
52.7 %
3.8 %
58.6 %
44.2 %
28.3%
R2
Counts
FL14-Width
FL5-Width

SSEA-1

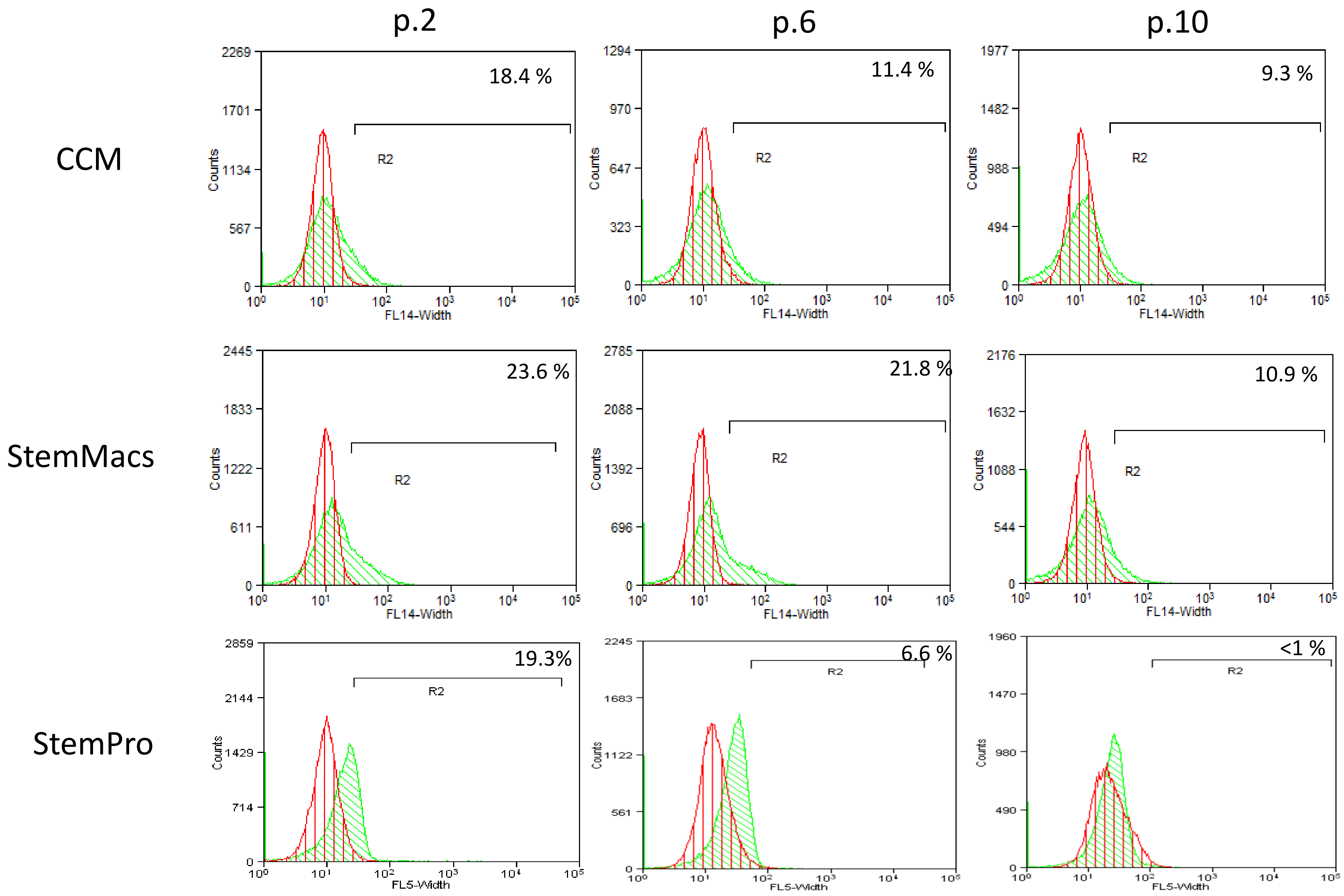
p.2
p.6
p.10
CCM
StemMacs
StemPro
18.4 %
11.4 %
9.3 %
23.6 %
21.8 %
10.9 %
19.3%
6.6 %
<1 %
R2
Counts
FL14-Width
FL5-Width

CD34

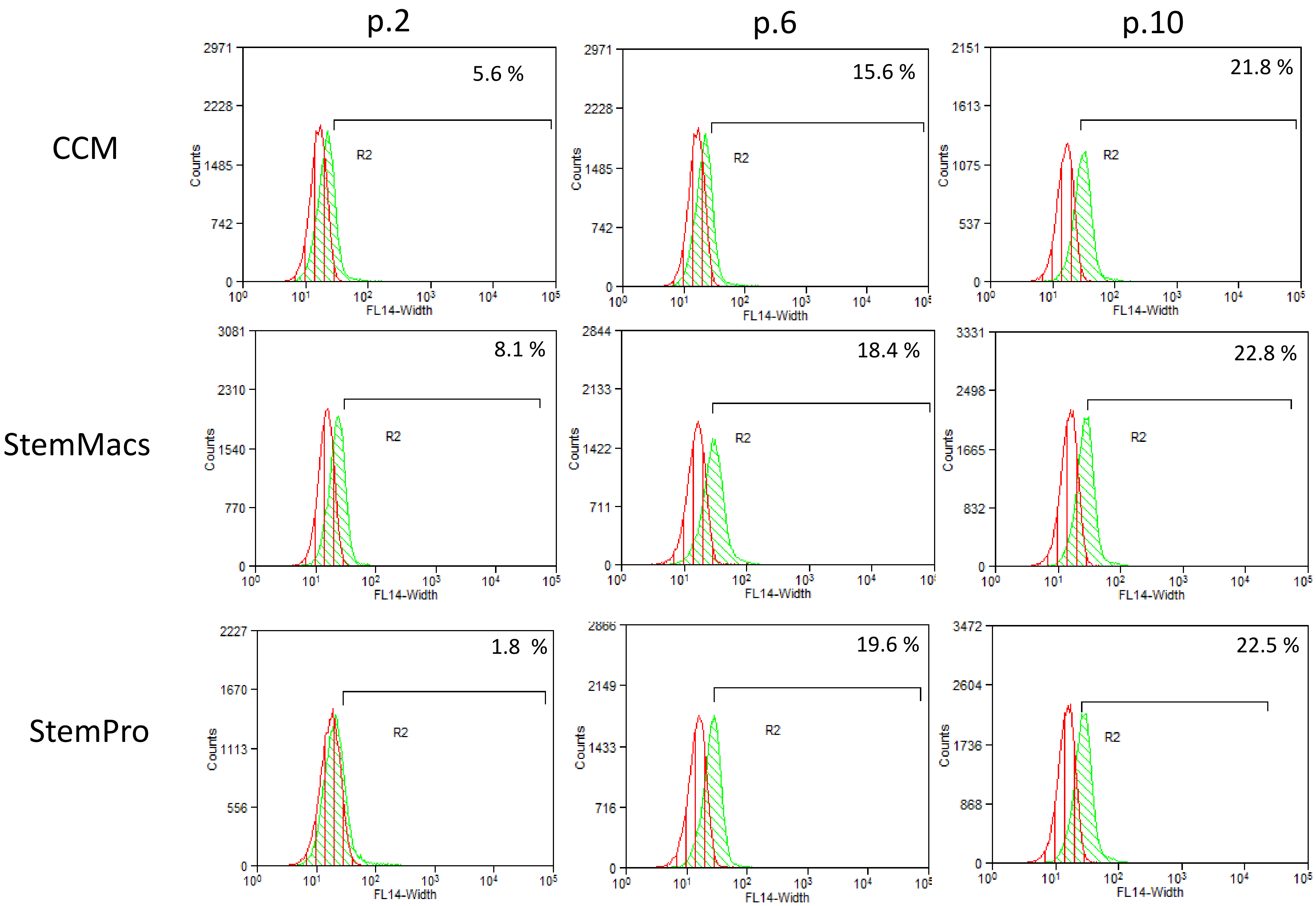
p.2
p.6
p.10
CCM
StemMacs
StemPro
5.6 %
15.6 %
21.8 %
8.1 %
18.4 %
22.8 %
1.8 %
19.6 %
22.5 %
R2
Counts
FL14-Width
